# Effects of Weight Loss Interventions on Gut Microbiota‐Derived Metabolites Linked to Cardiometabolic Health: A Systematic Review and Meta‐Analysis

**DOI:** 10.1111/obr.70151

**Published:** 2026-04-28

**Authors:** Fernando Q. Iorra, Leonardo André Swarowsky Loebens, Andrea Auler, Eduarda Caroline Tasso Nascimento, Beatriz D. Schaan, Patrícia Martins Bock

**Affiliations:** ^1^ Postgraduate Program in Medical Sciences: Endocrinology Universidade Federal do Rio Grande do Sul Porto Alegre Brazil; ^2^ School of Medicine Universidade Federal do Rio Grande do Sul Porto Alegre Brazil; ^3^ Post‐Graduate Program in Pharmacology and Therapeutics Universidade Federal do Rio Grande do Sul Porto Alegre Brazil; ^4^ Hospital de Clínicas de Porto Alegre Porto Alegre Brazil; ^5^ Post‐Graduate Program in Physiology Universidade Federal do Rio Grande do Sul Porto Alegre Brazil

**Keywords:** lipopolysaccharides, microbiome, obesity, secondary bile acids, short‐chain fatty acids, trimethylamine N‐oxide, weight management

## Abstract

**Background:**

Gut microbiota‐derived metabolites are emerging as potential mediators between obesity and cardiometabolic conditions. While weight loss interventions modify microbiota composition, their effects on systemic microbial metabolites remain unclear.

**Objective:**

To evaluate the effect of weight loss interventions on circulating levels of key gut‐derived metabolites in individuals with overweight or obesity, through a systematic review and meta‐analysis.

**Methods:**

Searches were performed in MEDLINE, EMBASE, and The Cochrane Library up to July 17, 2024. We included intervention studies in individuals with overweight/obesity undergoing weight loss strategies, with measurement of serum lipopolysaccharides (LPSs), trimethylamine N‐oxide (TMAO), secondary bile acids, short‐chain fatty acids (SCFAs), or indole derivatives. Meta‐analysis used the standardized mean difference (SMD) with a random‐effects model, stratified by intervention type (non‐surgical or surgical).

**Results:**

Sixty‐seven studies were included. Non‐surgical interventions were associated with reduced TMAO (*n* = 8 studies; SMD: –0.56; 95% CI –0.90 to −0.22; *I* ^2^ = 74.7%), but not LPS (*n* = 10 studies; SMD: –0.11; 95% CI –0.58 to 0.35; *I* ^2^ = 91.2%). Surgical interventions were associated with decreased LPS (*n* = 7 studies; SMD: –0.47; 95% CI –0.80 to −0.14; *I* ^2^ = 78.8%), and increased TMAO (*n* = 8 studies; SMD: 0.49; 95% CI 0.08 to 0.90; *I* ^2^ = 81.8%) and secondary bile acids (*n* = 13 studies; SMD: 0.48; 95% CI 0.30 to 0.66; *I* ^2^ = 58.8%). Evidence on SCFAs and indole derivatives was limited.

**Conclusion:**

Weight loss strategies have distinct effects on gut microbiota‐derived metabolites, underscoring the complexity of microbiota–host interactions and the potential for personalized approaches to obesity treatment.

## 1 Introduction

Obesity is a chronic condition characterized by excess adiposity that has reached pandemic proportions and has a significant impact on human health [1]. Its etiology is complex and involves genetic, behavioral, and environmental factors, a puzzle in which the gut microbiota has been recognized as a key piece [2, 3]. This microbial community influences energy extraction from food and energy storage in the host [4, 5], and generates metabolites with both local and systemic effects. These metabolites extend beyond energy homeostasis, potentially linking excess weight to obesity‐related disorders, including cardiovascular disease (CVD), diabetes, and metabolic‐associated steatotic liver disease [6].

Among the wide variety of gut microbiota metabolites, lipopolysaccharides (LPSs) and trimethylamine N‐oxide (TMAO) are of particular interest due to their potential association with cardiometabolic diseases [7]. The evidence suggests a possible association between these factors and excess weight, which could contribute to the development of atherosclerosis [8, 9, 10]. LPS, a component of gram‐negative bacterial cell walls, has the potential to enter the systemic circulation through intestinal barrier leakage, promoting toll‐like receptors (TLRs) activation and consequently low‐grade inflammation and pro‐atherogenic effects [11, 12]. Similarly, TMAO, generated from TMA by bacterial metabolism of dietary precursors such as choline and L‐carnitine, can also influence the formation of macrophage foam cells, platelet activation, and induction of endothelial dysfunction and inflammation [13].

Conversely, metabolites such as secondary bile acids, short‐chain fatty acids (SCFAs), and indole derivatives play crucial roles in maintaining intestinal barrier integrity, modulating inflammation, and regulating energy metabolism [14, 15]. Secondary bile acids originate from the transformation of host bile acids by the gut microbiota, with deoxycholic acid (DCA), lithocholic acid (LCA), and ursodeoxycholic acid (UDCA) as their unconjugated forms [16]. They can act as signaling molecules, activating farnesoid X receptors (FXRs) and G‐protein‐coupled receptor (TGR5) [17], which are expressed by intestinal L‐cells to promote GLP‐1 release [16, 18]. SCFAs, with an emphasis on acetate, butyrate, and propionate, are products of carbohydrate fermentation, influencing glucose and lipid metabolism as well as appetite control by stimulating the secretion of peptides such as GLP‐1 and PYY [19, 20]. Indole derivatives are mainly products of bacterial tryptophan catabolism, functioning as ligands of the aryl hydrocarbon receptor (AhR), with signaling actions such as reducing intestinal permeability, inducing GLP‐1 release, and modulating the immune response. Indole‐3‐lactate (ILA), indole‐3‐propionic acid (IPA), and indole‐3‐acetate (IAA) are among their main types [21].

While gut microbiota‐derived metabolites play important roles in both health and disease, the impact of weight loss interventions on their levels remains unclear. Increasing evidence suggests that weight loss strategies can alter gut microbiota composition [22, 23, 24, 25], but the effects of these interventions on gut‐derived metabolites are still not well understood. Interestingly, shifts in the microbiota may not necessarily result in the expected modifications in their derived metabolites, because studies focusing on the proportion of specific strains may not detect changes in less prevalent groups that could substantially contribute to metabolite modifications. Moreover, the presence of a particular phylum does not directly reflect its metabolic activity or its interactions with other microorganisms, diet, and host factors [15, 26]. Therefore, this systematic review seeks to assess how weight loss interventions—whether lifestyle, pharmacological, or surgical—influence gut microbiota‐derived metabolites in subjects with overweight or obesity, aiming to uncover potential mechanistic differences tied to microbiota modulation by each strategy.

## 2 Methods

This report follows the Preferred Reporting Items for Systematic Reviews and Meta‐Analyses guideline [27]. The protocol was registered on PROSPERO (CRD 420251012026).

### 2.1 Eligibility Criteria

We included intervention studies, whether randomized or not, conducted in people with overweight or obesity, undergoing weight loss interventions of any nature, either non‐surgical or surgical. Non‐surgical interventions encompassed a range of strategies, including lifestyle changes, dietary modifications, physical activity programs, and pharmacological treatments, implemented individually or in combination, provided they were part of a structured weight loss approach. Given the diversity of dietary interventions and their potential lack of specificity for weight loss, studies focusing solely on dietary interventions were included as long as they implemented a caloric deficit, regardless of diet composition or type. Studies involving the use of supplements, probiotics, prebiotics, or synbiotics alongside a structured weight loss strategy were also included. Surgical interventions referred to bariatric procedures, such as Roux‐en‐Y gastric bypass (RYGB), sleeve gastrectomy (SG), or related techniques.

The assessment of at least one of the metabolites of interest from the gut microbiota was incorporated into the search, including serum concentrations of LPS, TMAO, secondary bile acids, SCFAs, or indole derivatives. Studies were included if they provided sufficient data to calculate the change in levels of the biomarkers from pre‐ to post‐intervention. For studies that measured metabolites during fasting and after a meal tolerance test, we chose to extract baseline data by default, and those with measurements exclusively after a meal tolerance test were excluded. Case reports and studies conducted in animals or in vitro were excluded. Additionally, we also excluded studies that had performed only fecal or urinary dosage of the metabolites of interest. No language restrictions were established. We did not include conference abstracts.

### 2.2 Information Sources, Search Strategy, and Study Selection

We searched MEDLINE/PubMed, EMBASE, and The Cochrane Library, from inception to July 17, 2024, according to a structured search strategy specific to each database (Supporting Information, pages 1 and 2). The libraries were merged in Zotero® (v. 6.0.36), after which duplicates were removed. The final library was then exported to Rayyan® [28] for eligibility screening. The eligibility assessment by titles and abstracts was evaluated independently by pairs (FI and EN, LS and AA), and conflicts were resolved by consensus or by a third investigator (PB). Articles selected for full text were evaluated by the same peers, with disagreements also resolved by consensus or by a third investigator (PB).

### 2.3 Data Collection Process

Data extraction was independently performed in duplicate (FI and EN, LS and AA) in a Google Drive spreadsheet, collecting information on the date of publication, title, author, year of publication, study design, study primary and secondary outcomes, participant characteristics (mean age, sex), weight loss strategy, body mass index (BMI), and quantification of metabolites of interest pre‐ and post‐intervention. When presented, delta weight was extracted, or if unavailable, calculated based on pre‐ and post‐intervention BMI.

For TMAO and LPS, we extracted the total values. For secondary bile acids, we also extracted their total values, and when available, their unconjugated fractions (DCA, LCA, and UDCA). For SCFAs, we extracted data for the main fractions, namely, acetate, butyrate, and propionate. For indole derivatives, we collected data for ILA, IPA, and IAA, which are among the most frequently reported in the literature.

For studies with multiple follow‐up periods, the results from the longest follow‐up period available were extracted. If data were missing or results were presented only in graph form, the authors were contacted to provide the original numerical data. If a response was not obtained, those that presented graphical data were included in a software (WebPlotDigitizer, v 5.2, https://automeris.io) for data extraction. In crossover randomized trials, only pre‐crossover data were analyzed.

### 2.4 Study Risk of Bias Assessment

The risk of bias assessment was performed independently by two pairs of reviewers (FI and EN, LS and AA). Disagreements between reviewers were resolved through consensus or, when necessary, by a third reviewer (PB). For randomized controlled trials (RCTs), the RoB 2 (Risk of Bias 2) tool was used, while for non‐randomized intervention studies, the ROBINS‐I (Risk of Bias in Non‐randomized Studies of Interventions) tool was applied. Both tools provide a detailed and systematic evaluation of potential biases across various domains, such as randomization, blinding, and data analysis.

### 2.5 Data Synthesis and Statistical Analysis

Whenever necessary, extracted data were converted to mean and standard deviation. An initial attempt was made to harmonize biomarker units across studies using conversion formulas based on the molar mass of each metabolite. However, substantial variability in absolute biomarker values remained despite this standardization, likely due to methodological differences (e.g., assay types, sample handling). Thus, although the original protocol planned to use the weighted mean difference (WMD), we opted to use the standardized mean difference (SMD) for each metabolite instead.

SMDs were derived from within‐group changes (post‐minus pre‐intervention). Hedges' *g* and corresponding standard errors were calculated using the *escalc* function from the *metafor* package in R (v4.5.0), with the SMCC (standardized mean change with assumed correlation) metric. A pre–post correlation of *r* = 0.5 was assumed. A random‐effects model was applied for the meta‐analysis, and the results were presented in forest plots. Heterogeneity was assessed using *I* ^2^, and potential publication bias was evaluated by visual inspection of funnel plots.

All analyses were stratified by type of intervention (surgical vs. non‐surgical), as detailed in the eligibility criteria. In studies with more than one arm containing weight loss interventions of the same nature (e.g., two surgical techniques for weight loss or two types of low‐calorie diet), the results were combined for these groups, according to the formula available in the Cochrane handbook [29], and then analyzed as a single intervention. To explore sources of heterogeneity, sensitivity analyses were conducted according to the degree of weight loss (excluding studies with < 5% weight reduction), and risk of bias (excluding studies with high risk of bias). An additional sensitivity analysis within the non‐surgical group was conducted by restricting to dietary interventions only, excluding studies with co‐interventions (e.g., supplements) or those based on physical activity or pharmacological agents. For surgical interventions, a subgroup analysis was performed by surgical technique, separating study arms that had been pooled in the main analysis.

## 3 Results

### 3.1 Study Selection and Characteristics of Included Studies

The study selection process is shown in Figure 1. After removing duplicates, 4646 studies underwent title and abstract selection, with 186 retained for full‐text analysis. Of these, 67 were included, 16 being of LPS [30, 31, 32, 33, 34, 35, 36, 37, 38, 39, 40, 41, 42, 43, 44, 45], 15 of TMAO [13, 33, 35, 46, 47, 48, 49, 50, 51, 52, 53, 54, 55, 56, 57], 30 of secondary bile acids [57, 58, 59, 60, 61, 62, 63, 64, 65, 66, 67, 68, 69, 70, 71, 72, 73, 74, 75, 76, 77, 78, 79, 80, 81, 82, 83, 84, 85, 86], seven of SCFAs [51, 87, 88, 89, 90, 91, 92], and three of indole derivatives [93, 94, 95]. Four studies analyzed two metabolites of interest simultaneously.

**FIGURE 1:**
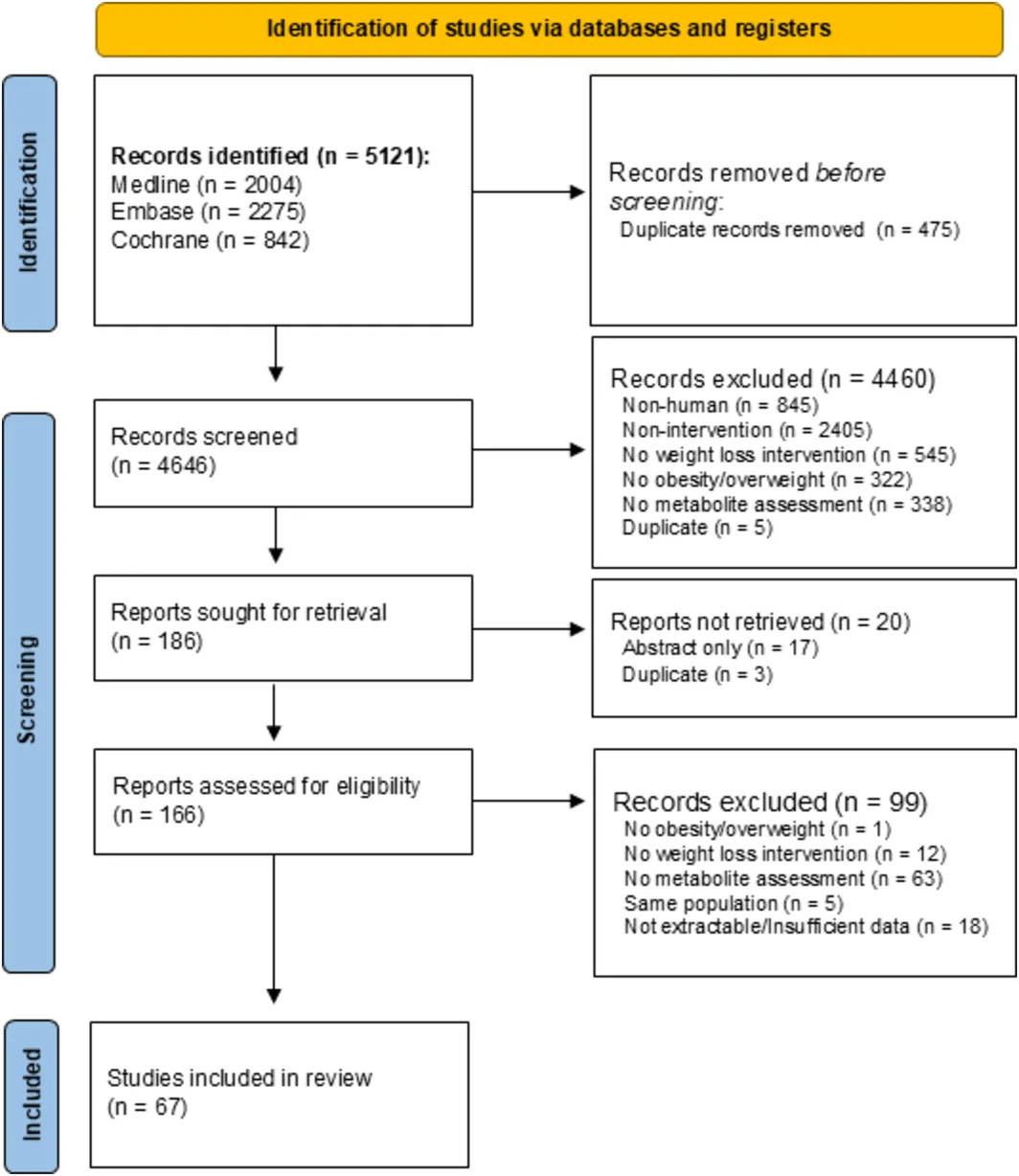
PRISMA flow diagram of included studies (Adapted from Page MJ, et al. BMJ 2021;372:n71).

The characteristics of the included studies, such as details of the design, intervention, population, and outcome, are shown in Table S1 (Supporting Information, pages 3 to 8). Of the 67 included studies, 18 were RCTs, 47 were non‐randomized studies of intervention (NRSI), and 2 studies contained both designs, which were included separately. Detailed reasons for exclusion of each article assessed at the full‐text stage are provided in Supplementary Table S2 (Supporting Information, pages 15 to 21).

### 3.2 Risk of Bias and Publication Bias Assessment

For randomized clinical trials, the RoB 2 tool was used. Overall, most studies were rated as having some concerns (*n* = 52.4%), primarily due to bias in the selection of the reported result (some concerns in 47.6%) or issues with the randomization process (some concerns in 42.9%). For non‐randomized studies, the ROBINS‐I tool was applied. The overall risk of bias was high (between 50% and 75%), mainly due to concerns in the confounding domain. A detailed risk of bias assessment is provided in the Supporting Information (Figures S1–S4).

Regarding publication bias, given the analysis stratified by type of intervention (surgical vs. non‐surgical), few studies remained in each subgroup. Although funnel plots were visually inspected, the small number of studies per group limited the ability to reliably assess small‐study effects. For the same reason, Egger's test was not performed.

### 3.3 Synthesis of Results

#### 3.3.1 Lipopolysaccharide

Among the studies evaluating LPS, nine involved non‐surgical interventions and six focused on surgical strategies. One study included both intervention types; its arms were analyzed separately and labeled as NS (non‐surgical) and S (surgical). Overall, non‐surgical interventions had no significant effect on LPS levels (SMD –0.11; 95% CI –0.58 to 0.35; *I* ^2^ = 91.2%; Figure 2A). These interventions comprised different hypocaloric dietary approaches (low‐calorie, ketogenic, DASH, and intermittent fasting diets, as well as a diet combined with probiotic supplementation), typically involving a ~ 500 kcal/day energy deficit, exercise‐based programs, and structured medical care including diet recommendation and pharmacologic management. Given the heterogeneity of intervention types, subgroup analyses were conducted restricting the sample to dietary‐only studies (without co‐interventions such as supplements or exercise), but the results remained largely unchanged. Sensitivity analyses using delta BMI and risk of bias were also performed (Table S3).

**FIGURE 2:**
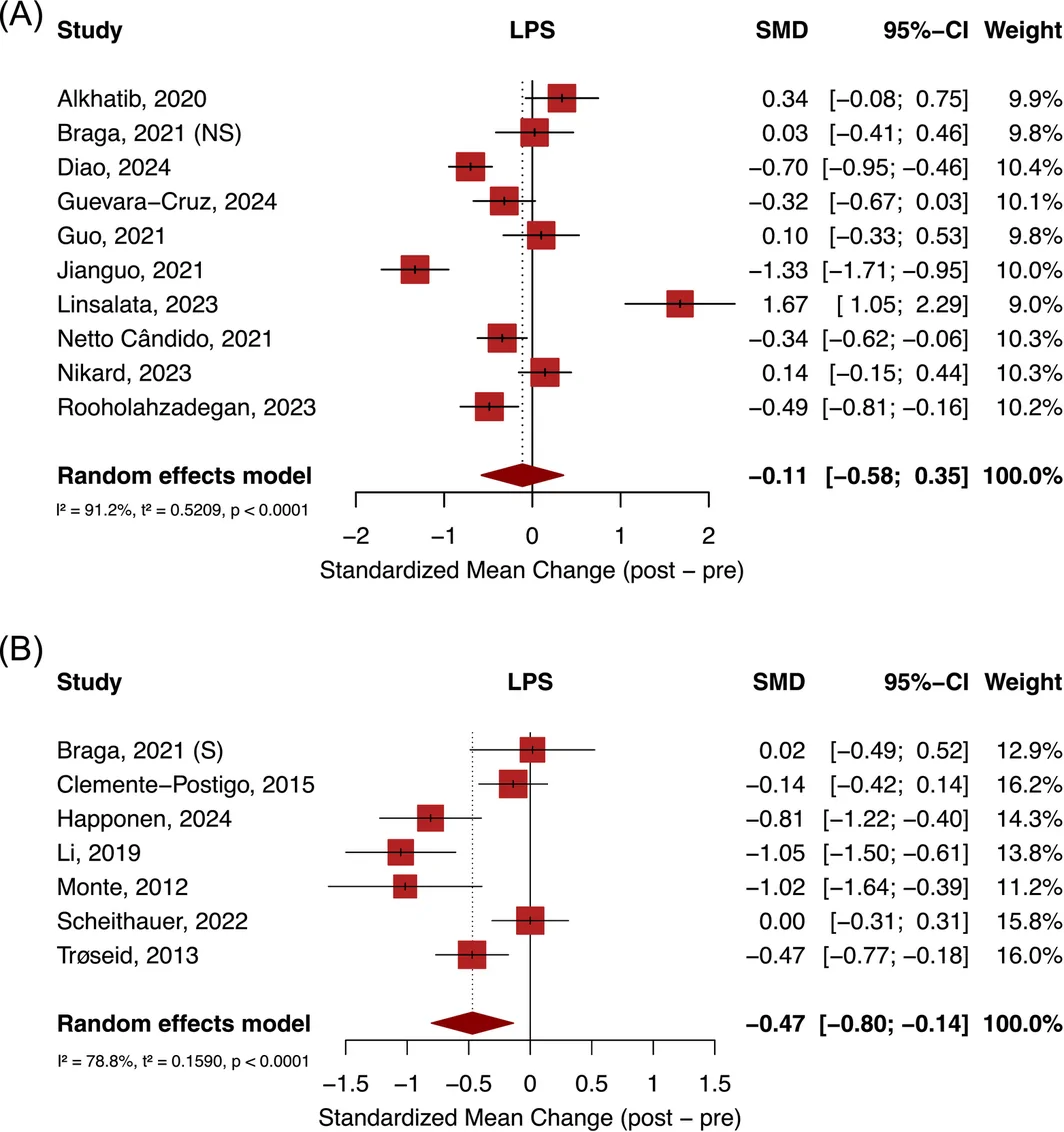
Effect of weight loss interventions on LPS levels. (A) Non‐surgical weight loss interventions. (B) Surgical weight loss interventions. *Legend: (S) = surgical arm; (NS) = non‐surgical arm*.

Conversely, surgical interventions were associated with a significant reduction in LPS (SMD –0.47; 95% CI –0.80 to −0.14; *I* ^2^ = 78.8%; Figure 2B). Stratification by surgical technique indicated consistent reductions in the subgroup combining RYGB, duodenal switch (DS), and biliopancreatic diversion (BPD) studies (*n* = 6; SMD –0.37; 95% CI –0.70 to −0.04; *I* ^2^ = 72.9%). Only two studies [32, 38] reported isolated results for SG, precluding meta‐analysis, although both showed significant reductions in LPS levels. Heterogeneity remained high in sensitivity analyses stratified by delta BMI and risk of bias (Table S4).

#### 3.3.2 Trimethylamine N‐Oxide

Seven studies evaluated non‐surgical interventions targeting TMAO levels, seven assessed surgical interventions, and one included both approaches (arms labeled as NS and S, respectively). Non‐surgical interventions were associated with a reduction in TMAO levels (SMD –0.56; 95% CI –0.90 to −0.22; *I* ^2^ = 74.7%; Figure 3A). Interventions included different dietary regimens (low‐calorie, DASH, ketogenic, and intermittent fasting) and lifestyle‐based programs combining exercise with caloric restriction. Most protocols involved an energy deficit of 500–800 kcal/day. One study assessed a pharmacological intervention (liraglutide). To improve grouping consistency, a subanalysis excluding studies with co‐interventions other than hypocaloric diets was performed. Additional subgroup analyses by delta BMI and risk of bias were also conducted, but none materially altered the effect estimates or reduced heterogeneity (Table S5).

**FIGURE 3:**
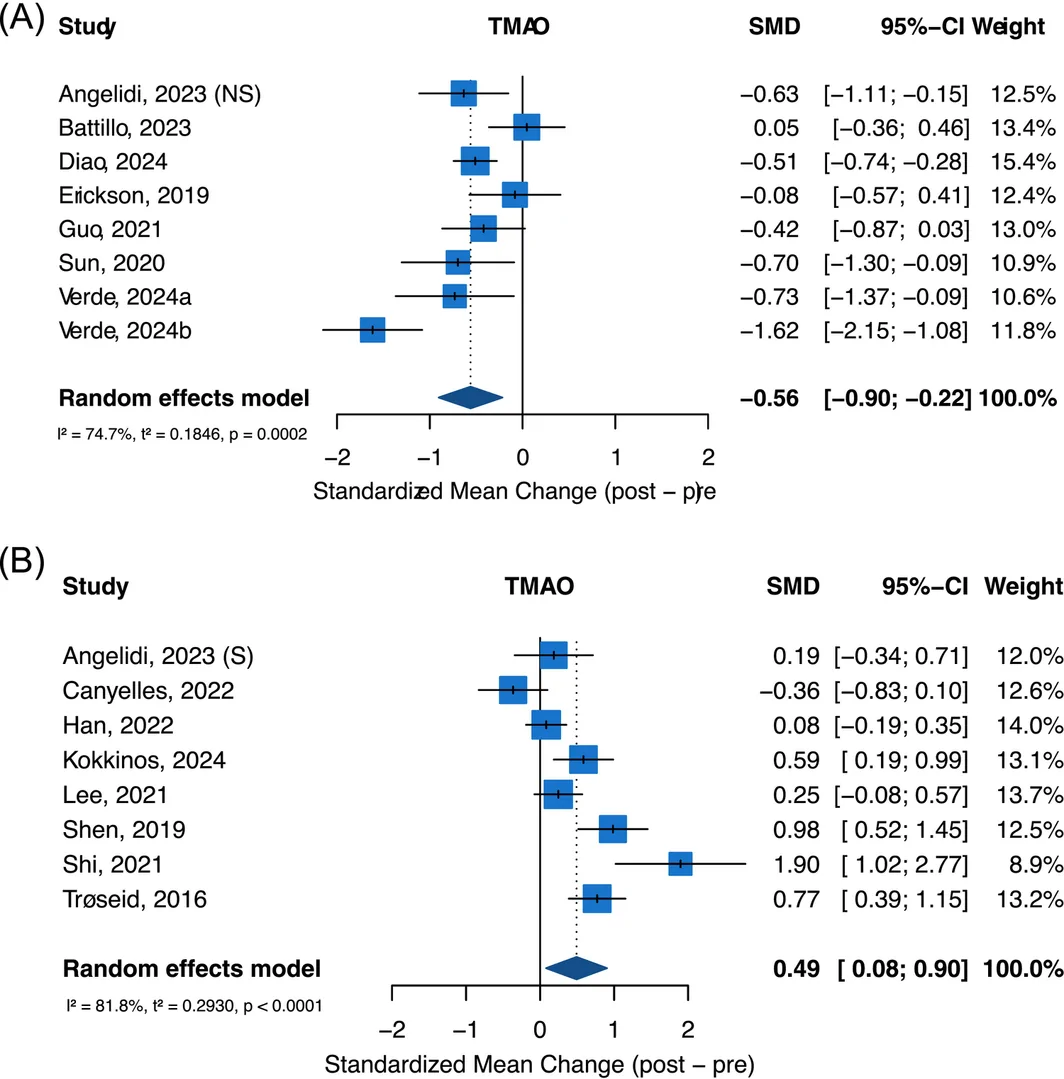
Effect of weight loss interventions on TMAO levels. (A) Non‐surgical weight loss interventions. (B) Surgical weight loss interventions. *Legend: (S) = surgical arm; (NS) = non‐surgical arm*.

On the other hand, surgical interventions were associated with an increase in TMAO levels (SMD 0.49; 95% CI 0.08 to 0.90; *I* ^2^ = 81.8%; Figure 3B). When stratified by surgical technique, this finding was consistent in studies including RYGB or DS, which showed increased TMAO levels (*n* = 5; SMD 0.81; 95% CI 0.28 to 1.34; *I* ^2^ = 69.4%). In contrast, only three studies reported individual results for SG, none of which showed significant changes in TMAO levels; accordingly, the pooled analysis for this subgroup, although limited by the small number of studies, also showed no significant effect (*n* = 3; SMD 0.14; 95% CI –0.15 to 0.43; *I* ^2^ = 0%). Further stratification by delta BMI and risk of bias removed statistical significance, while heterogeneity remained high (Table S6).

#### 3.3.3 Secondary Bile Acids

One study investigated the effects of non‐surgical interventions on secondary bile acids, 12 studies assessed surgical interventions, and one study included both types of interventions. Due to the limited number of non‐surgical studies, a meta‐analysis was not conducted for this subgroup. Palomatti et al. [75] evaluated a lifestyle‐based intervention, and Sachdev et al. [77] assessed intensive medical management; in both, no significant changes in secondary bile acids were observed.

In contrast, surgical interventions were associated with an increase in secondary bile acid levels (SMD 0.48; 95% CI 0.30 to 0.66; *I* ^2^ = 58.8%; Figure 4). Stratified analysis by surgical technique showed consistent results when pooling studies of RYGB, omega‐loop gastric bypass (OLGB), BPD with duodenal switch (BPD/DS), and laparoscopic sleeve gastrectomy with duodenojejunal bypass (LSG/DJB) (*n* = 11; SMD 0.47; 95% CI 0.29 to 0.66; *I* ^2^ = 49.9%). Furthermore, restricting the analysis to RYGB studies alone yielded a notable reduction in heterogeneity (*n* = 8; SMD 0.51; 95% CI: 0.34 to 0.68; *I* ^2^ = 26.2%). Conversely, the subgroup analysis of SG and laparoscopic adjustable gastric banding (LAGB) studies showed no significant association (*n* = 7; SMD 1.47; 95% CI: −0.85 to 3.78; *I* ^2^ = 89.7%), and this finding remained unchanged when only SG studies were included (*n* = 4; SMD 2.45; 95% CI: −1.89 to 6.79; *I* ^2^ = 94.6%). Sensitivity analysis for delta BMI was not performed, as all surgical studies reported weight loss greater than 5% (Table S7).

**FIGURE 4:**
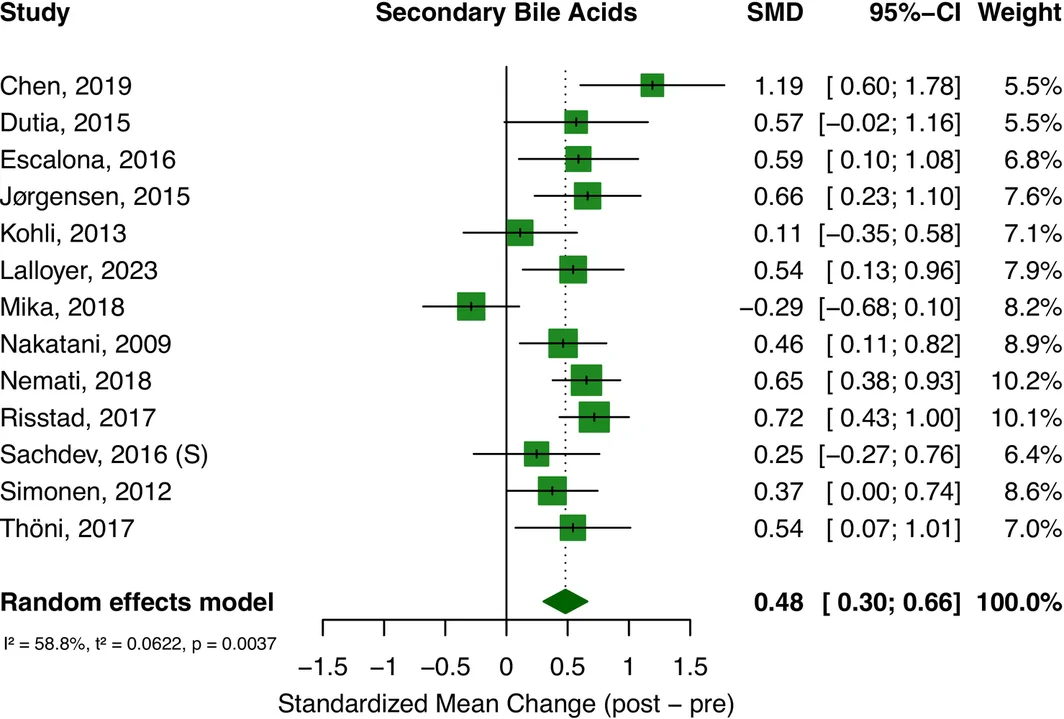
Effect of surgical weight loss interventions on total secondary bile acid levels. *Legend: (S) = surgical arm*.

In the analysis of unconjugated fractions of secondary bile acids, non‐surgical interventions were associated with a reduction in UDCA (Supporting Information, Figure S3A). However, these interventions did not significantly affect DCA (Supporting Information, Figure S5A). Only three non‐surgical studies evaluated LCA; therefore, we did not perform a meta‐analysis for this outcome. Additionally, surgical interventions did not lead to significant changes in any of the unconjugated fractions evaluated (Supporting Information, Figures S3B, S4A, and S5B).

#### 3.3.4 Short‐Chain Fatty Acids

In the SCFAs studies, non‐surgical interventions demonstrated very low consistency among themselves, and the number of studies was very limited (*n* = 3). Therefore, no meta‐analysis was conducted for this outcome. Among the included studies, Iversen et al. [88] reported an increase in plasma butyrate after 12 weeks of a hypocaloric diet including high‐fiber rye products compared with refined wheat, while other SCFAs remained unchanged. Yang et al. [85] observed an increase in butyrate, but not in acetate or propionate, following a 24‐week intervention combining a hypocaloric diet and a weight maintenance phase. In contrast, Tilves et al. [90] found no significant changes in acetate, propionate, or butyrate after 1 year of a lifestyle‐based weight loss intervention.

Regarding surgical interventions, the number of studies was also limited (*n* = 4), and a narrative synthesis is presented. Ahmad et al. [87] evaluated the effect of SG, and Shen et al. [51] evaluated the effect of either SG or RYGB. Both studies found no significant changes in acetate, butyrate, or propionate levels. Two other studies evaluated the effects of RYGB on SCFAs, with conflicting results. Martínez‐Sánchez et al. [89] found a decrease in acetate and an increase in butyrate and propionate, whereas Wijayatunga et al. [91] observed stable acetate and a reduction in butyrate concentrations.

#### 3.3.5 Indole Derivatives

Three studies examined serum indole derivative levels, with two utilizing surgical weight loss interventions and one employing dietary intervention. Due to the limited number of studies and methodological heterogeneity, a meta‐analysis was not feasible. The dietary study [95] found no significant changes in IPA or IAA levels. Among the surgical studies, Ahlin et al. [93] reported an increase in IAA 6 months after RYGB or BPD, whereas Kwon et al. [94] observed a decrease in ILA 3 months after SG, with no significant changes in IPA or IAA.

## 4 Discussion

This systematic review and meta‐analysis provide a comprehensive synthesis of current evidence on how weight loss interventions influence gut microbiota‐derived metabolites in individuals with overweight or obesity. Given the interconnected roles of gut‐derived metabolites in metabolic health, we adopted a broader approach to synthesize evidence across multiple metabolite classes. This strategy aims to capture overall patterns of response to weight loss interventions, acknowledging that different strategies may influence distinct pathways simultaneously. Overall, the findings reveal metabolite‐specific and intervention‐specific effects, underscoring the complexity of host‐microbiota interactions in the context of weight reduction.

Among the five classes of metabolites assessed, LPS and TMAO—both of which are potentially associated with metabolic and cardiovascular risk [7]—demonstrated divergent responses depending on the intervention type. LPS levels did not change significantly after non‐surgical interventions, most of which involved a low‐calorie diet. In contrast, TMAO levels decreased after this type of intervention. Although dietary changes, even in the short term, are known to influence the gut microbiota [96], the reduction in TMAO observed in our study can primarily reflect decreased caloric intake, which would reduce the availability of dietary precursors such as choline and carnitine without necessarily impacting LPS levels. Given that a systematic review of trials is not designed to disentangle the relative contributions of reduced precursor intake, microbiota shifts, or other physiological adaptations, mechanistic inferences should be approached with caution.

Surgical interventions were also associated with distinct outcomes for LPS and TMAO levels. Bariatric surgery led to a significant reduction in circulating LPS, most consistently after RYGB, DS, and BPD, likely reflecting improved gut barrier integrity and decreased systemic endotoxemia, both of which are commonly impaired in obesity and type 2 diabetes. Anatomical and physiological changes induced by surgery may reduce intestinal permeability and reshape the microbiota, limiting the translocation of LPS‐producing Gram‐negative bacteria or their components into the bloodstream [97]. Notably, Torsten et al. proposed that enhanced intestinal IgA secretion following bariatric surgery plays a key role in reinforcing mucosal immunity and preventing endotoxin leakage [44], a mechanism that may underlie the postoperative improvements in inflammation and metabolic health.

Conversely, bariatric surgery was associated with increased levels of circulating TMAO, which was also most consistently observed after RYGB and DS. This modification may be related to changes in the gut microbiota, but also to intestinal transit or other physiological adaptations. Interpretation based on microbial composition is limited, as genes involved in TMA production (such as *cutC* and *cntA*) can be detected in human fecal samples, yet the majority of *cutC*‐positive sequences exhibit low similarity to reference genomes, suggesting that key TMA‐producing bacteria remain largely uncharacterized [98]. In contrast, hepatic conversion of TMA to TMAO presents a more plausible explanatory pathway. Experimental evidence from animal models has shown that FMO‐3, the main enzyme responsible for this conversion, is upregulated by the FXR, which is activated by bile acids [99]. Given the observed postoperative increase in secondary bile acid concentrations in our study, it is plausible to consider the hypothesis that enhanced FXR signaling may induce FMO3 expression, thereby contributing to an elevation in TMAO levels.

Beyond LPS and TMAO, other gut‐derived metabolites also exhibited distinct response patterns following weight loss interventions. Secondary bile acids were consistently increased after surgical procedures, particularly RYGB. This result is in line with most studies in the literature and may be due to decreased fecal bile acid excretion and increased bile acid recirculation [100]. Exposure to gut microbiota provides opportunities for primary bile acids modification and diversification [101]. Therefore, increased secondary bile acids may reflect both changes in intestinal transit and microbiota. Regarding the measurement of subfractions, surgical interventions did not influence LCA, DCA, or UDCA individually. Nevertheless, this may be due to limited sensitivity, particularly for metabolites present at lower concentrations. Furthermore, postprandial bile acid dynamics, which may have some additional role, may not be captured under fasting conditions and would require a meal challenge to detect. Non‐surgical interventions were associated with reduced UDCA levels. However, the small number of studies and the broad range of non‐surgical interventions, often mixing diets and physical activity, could be confounding factors for this finding.

Conflicting results were observed regarding the effect of weight loss interventions on circulating SCFAs. This finding is most likely a consequence of the low number of studies and methodological heterogeneity. SCFAs exert local actions in the gut and portal circulation, and plasma levels may not accurately reflect their tissue‐specific functions or production dynamics [20]. Similarly, data on indole derivatives were too scarce and heterogeneous to allow any definitive interpretation, though preliminary evidence suggests context‐specific changes depending on the type of surgery or dietary modulation.

Another relevant point concerns the influence of the magnitude of weight loss on levels of microbiota‐derived metabolites. In the sensitivity analysis restricted to studies reporting ≥ 5% BMI reduction, there was no substantial modification of the results or improvement in heterogeneity. This finding suggests that the extent of weight loss alone may not be the primary driver of the gut‐derived metabolomic changes observed after bariatric surgery. Supporting this, previous research has shown that the gut microbiota of women who underwent RYGB or vertical banded gastroplasty differed significantly from that of non‐operated women with similar BMI. Moreover, the microbiota profiles of non‐operated women and an independent control group were highly similar, reinforcing the idea that the microbiota changes observed post‐surgery are not merely a consequence of reduced adiposity [102]. Instead, bariatric surgery appears to induce specific and persistent shifts in gut microbiota composition and function, such as increased *Proteobacteria* and decreased clostridial species [24, 102], likely reflecting direct effects of the surgical intervention on the gut microbial ecosystem.

### 4.1 Biological Implications

The observed reduction in LPS following surgical interventions aligns with previous findings suggesting improved gut barrier function and decreased systemic endotoxemia after bariatric surgery [44, 97]. Given the role of LPS in TLR4‐mediated activation of innate immunity and low‐grade chronic inflammation, this reduction may contribute to the well‐established anti‐inflammatory and insulin‐sensitizing effects of bariatric procedures [103]. Notably, the absence of a similar reduction in LPS levels after non‐surgical strategies, despite meaningful weight loss in several studies, suggests that the microbial and epithelial remodeling induced by surgery may not be fully replicated by dietary or behavioral interventions alone.

Considering the expected positive effect of weight loss on cardiovascular risk, the increase in TMAO levels due to surgical interventions is intriguing. This finding may seem counterintuitive in the context of presumed improvements in metabolic health. However, it is important to recognize that TMAO alone may have limited predictive value in certain settings [104, 105], raising the possibility that its elevation represents a byproduct of altered physiology rather than a direct mediator of harm in the post‐bariatric context.

The consistent increase in secondary bile acids, particularly after surgical interventions, may help mediate the metabolic benefits of bariatric surgery. These bile acids can activate receptors such as FXR and TGR5 [17]. TGR5 influences lipid and carbohydrate metabolism, energy expenditure, and inflammatory pathways [106], while FXR plays a key role in glucose and lipid homeostasis. Both receptors are expressed in intestinal L‐cells, where their activation promotes GLP‐1 secretion [16, 18], contributing to appetite regulation and metabolic improvements.

### 4.2 Strengths and Limitations

To our knowledge, this is the first meta‐analysis to comprehensively evaluate the effect of weight loss interventions on multiple classes of gut‐derived metabolites, incorporating a broad spectrum of strategies and analytical methods, thereby providing a valuable synthesis of current evidence. Methodological strengths include protocol registration, rigorous risk of bias assessment, and detailed subgroup and sensitivity analyses.

However, it is important to acknowledge several limitations when interpreting our findings. First, interventions were broadly categorized as surgical or non‐surgical, despite substantial disparities in study designs, protocols, and interventions. Grouping different non‐surgical protocols or surgical techniques may have introduced bias. Mitigation efforts were made through subanalyses restricted to more homogeneous protocols (e.g., comparable dietary interventions or operative procedures) whenever feasible. Furthermore, the overall risk of bias was mostly moderate to high. These aspects likely contributed to the high heterogeneity observed across analyses, which was only partially addressed by the sensitivity analyses, limiting the interpretability of the findings. The paucity of studies evaluating SCFAs and indole derivatives has also hindered the establishment of robust conclusions regarding these groups. Finally, mechanistic inferences are inherently limited by the nature of our study and by the absence, in most primary studies, of data correlating gut microbiota composition with metabolomic outcomes. Future studies with greater methodological standardization are needed to elucidate the true impact of these interventions on the levels of metabolites derived from the gut microbiota.

## 5 Conclusion

Weight loss interventions exert distinct effects on gut microbiota‐derived metabolites depending on the strategy employed. These findings highlight the complexity of host–microbiota interactions during weight loss and suggest that the type of intervention plays a critical role in shaping gut‐derived metabolic profiles. Recognizing the metabolite‐specific responses to weight loss strategies can help identify biomarkers for monitoring intervention efficacy or for tailoring treatments to individual microbial and metabolic profiles.

## Funding

F.Q.I. received an educational scholarship grant from Coordenação de Aperfeiçoamento de Pessoal de Nível Superior—Brasil (CAPES).

## Conflicts of Interest

The authors declare no conflicts of interest.

## Supporting information


**Table S1:** Characteristics of included studies.

## Data Availability

Data generated and analyzed during this study are included in this published article or in the data repositories listed in References.
