# Supplementary material for: Effects of Weight Loss Interventions on Gut Microbiota‐Derived Metabolites Linked to Cardiometabolic Health: A Systematic Review and Meta‐Analysis
Source: Obes Rev. 2026 Apr 28;27(11):e70151. doi: 10.1111/obr.70151 (PMC13655986; doi:10.1111/obr.70151)
Supplement: Supplementary file 1 [file OBR-27-e70151-s001.pdf]

SUPPLEMENTARY MATERIAL

Main Paper: *Effects of Weight Loss Interventions on Gut Microbiota-Derived Metabolites Linked to Cardiometabolic Health: A Systematic Review and Meta-Analysis*

Authors: Fernando de Quadros Iorra, Leonardo André Swarowsky, Andrea Auler, Eduarda Caroline Tasso Nascimento, Beatriz D. Schaan, Patrícia Martins Bock

Corresponding author: fernando.iorra@ufrgs.br

Search strategy

**PUBMED - Medline**
(Obesity[mh] OR Obesit*[tiab] OR Overweight[mh] OR Overweight[tiab] OR weight excess[tiab])<br>**AND**<br>(Drug Therapy[mh] OR Drug Therap*[tiab] OR Pharmacotherap*[tiab] OR Weight Loss[mh] OR Weight Loss*[tiab] OR Weight Reduction*[tiab] OR Weight Reduction Programs[mh] OR Bariatric Surgery[mh] OR Bariatric Surg*[tiab] OR Metabolic Surger*[tiab] OR Stomach Stapling[tiab] OR Liraglutide[mh] OR Liraglutide[tiab] OR Victoza[tiab] OR Saxenda[tiab] OR NN 2211[tiab] OR NN2211[tiab] OR semaglutide[tiab] OR Rybelsus[tiab] OR Wegovy[tiab] OR Ozempic[tiab] OR tirzepatide[tiab] OR Glucagon-Like Peptide-1 Receptor Agonists[pa] OR Glucagon-Like Peptide-1 Receptor Agonists[tiab] OR sibutramine[nm] OR sibutramine[tiab] OR di-desmethylsibutramine[tiab] OR didesmethylsibutramine[tiab] OR Reductil[tiab] OR mono-desmethylsibutramine[tiab] OR Meridia[tiab] OR Phentermine[tiab] OR Topiramate[mh] OR Topiramate[tiab] OR Topamax[tiab] OR Orlistat[mh] OR Orlistat[tiab] OR Tetrahydrolipstatin[tiab] OR THLP[tiab] OR Tetrahydrolipastatin[tiab] OR Xenical[tiab] OR bupropion hydrochloride, naltrexone hydrochoride drug combination[nm] OR bupropion hydrochloride, naltrexone hydrochoride drug combination[tiab] OR Mysimba[tiab] OR Contrave[tiab] OR Diet Therapy[mh] OR Diet Therap*[tiab] OR Dietary Restriction*[tiab] OR Dietary Modification*[tiab] OR Diet Modification*[tiab])<br>**AND**<br>(trimethyloxamine[nm] OR trimethyloxamine[tiab] OR trimethylammonium oxide[tiab] OR trimethylamine N-oxide[tiab] OR TMAO[tiab] OR trimethylamine oxide[tiab] OR Bile Acids and Salts[mh] OR Bile Acid*[tiab] OR Bile Salt*[tiab] OR Fatty Acids, Volatile[mh] OR Volatile Fatty Acid*[tiab] OR Short Chain Fatty Acid*[tiab] OR Propionates[mh] OR Propionate*[tiab] OR Propanoate*[tiab] OR Propionic Acid*[tiab] OR Propanoic Acid*[tiab] OR Butyrates[mh] OR Butyrate*[tiab] OR Butyric Acids[tiab] OR Butanoic Acids[tiab] OR Acetates[mh] OR Acetate*[tiab] OR Acetic Acid*[tiab] OR Indoles[mh] OR Indol*[tiab] OR Indican[mh] OR Indican[tiab] OR Indoxyl Sulfate[tiab] OR indolepropionic acid[nm] OR Glucosyltransferase*[tiab] OR indole-3-acetate beta-glucosyltransferase[nm] OR IAGlc synthase[tiab] OR IAA-glucose synthetase[tiab] OR indole-3-carbaldehyde[nm] OR thiamine indole-3-propionate[nm] OR TI-3-P[tiab] OR Lipopolysaccharides[mh] OR Lipopolysaccharide*[tiab] OR Lipoglycans[tiab])

**EMBASE***
('obesity'/exp OR Obesit*:ti,ab,kw OR Overweight:ti,ab,kw OR 'weight excess':ti,ab,kw)<br>**AND**<br>('drug therapy'/exp OR 'Drug Therap*':ti,ab,kw OR Pharmacotherap*:ti,ab,kw OR 'body weight loss'/exp OR 'Weight Loss*':ti,ab,kw OR 'Weight Reduction*':ti,ab,kw OR 'weight loss program'/exp OR 'bariatric surgery'/exp OR 'Bariatric Surg*':ti,ab,kw OR 'Metabolic Surger*':ti,ab,kw OR 'Stomach Stapling':ti,ab,kw OR 'liraglutide'/exp OR Liraglutide:ti,ab,kw OR Victoza:ti,ab,kw OR Saxenda:ti,ab,kw OR 'NN 2211':ti,ab,kw OR NN2211:ti,ab,kw OR semaglutide:ti,ab,kw OR Rybelsus:ti,ab,kw OR Wegovy:ti,ab,kw OR Ozempic:ti,ab,kw OR Tirzepatide:ti,ab,kw OR 'glucagon like peptide 1 receptor agonist'/exp OR 'Glucagon-Like Peptide-1 Receptor Agonists':ti,ab,kw OR 'sibutramine'/exp OR sibutramine:ti,ab,kw OR 'di-desmethylsibutramine':ti,ab,kw OR didesmethylsibutramine:ti,ab,kw OR Reductil:ti,ab,kw OR 'mono-desmethylsibutramine':ti,ab,kw OR Meridia:ti,ab,kw OR Phentermine:ti,ab,kw OR 'topiramate'/exp OR Topiramate:ti,ab,kw OR Topamax:ti,ab,kw OR 'orlistat'/exp OR Orlistat:ti,ab,kw OR Tetrahydrolipstatin:ti,ab,kw OR THLP:ti,ab,kw OR Tetrahydrolipastatin:ti,ab,kw OR Xenical:ti,ab,kw OR 'bupropion hydrochloride, naltrexone hydrochoride drug combination':ti,ab,kw OR Mysimba:ti,ab,kw OR Contrave:ti,ab,kw OR 'diet therapy'/exp OR 'Diet Therap*':ti,ab,kw OR 'Dietary Restriction*':ti,ab,kw OR 'Dietary Modification*':ti,ab,kw OR 'Diet Modification*':ti,ab,kw)<br>**AND**<br>('trimethyloxamine'/exp OR 'trimethyloxamine':ti,ab,kw OR 'trimethylammonium oxide':ti,ab,kw OR 'trimethylamine N-oxide':ti,ab,kw OR TMAO:ti,ab,kw OR 'trimethylamine oxide':ti,ab,kw OR 'bile acid'/exp OR 'Bile Acid*':ti,ab,kw OR 'Bile Salt*':ti,ab,kw OR 'volatile fatty acid'/exp OR 'Volatile Fatty Acid*':ti,ab,kw OR 'Short Chain Fatty Acid*':ti,ab,kw OR 'propionic acid derivative'/exp OR Propionate*:ti,ab,kw OR Propanoate*:ti,ab,kw OR 'Propionic Acid*':ti,ab,kw OR 'Propanoic Acid*':ti,ab,kw OR 'butyric acid derivative'/exp OR Butyrate*:ti,ab,kw OR 'Butyric Acids':ti,ab,kw OR 'Butanoic Acids':ti,ab,kw OR 'acetic acid derivative'/exp OR Acetate*:ti,ab,kw OR 'Acetic Acid*':ti,ab,kw OR 'indole derivative'/exp OR Indol*:ti,ab,kw OR 'indican'/exp OR Indican:ti,ab,kw OR

'Indoxyl Sulfate':ti,ab,kw OR 'indolepropionic acid'/exp OR Glucosyltransferase*:ti,ab,kw OR 'IAGlc synthase':ti,ab,kw OR 'IAA-glucose synthetase':ti,ab,kw OR 'indole 3 carboxaldehyde'/exp OR 'TI-3-P':ti,ab,kw OR 'lipopolysaccharide'/exp OR Lipopolysaccharide*:ti,ab,kw OR Lipoglycans:ti,ab,kw)<br>AND [embase]/lim NOT ([embase]/lim AND [medline]/lim)

* For an EMBASE database, we applied a filter to remove conference abstracts.

**The Cochrane Library**
([mh Obesity] OR [mh Overweight] OR (Obesit* OR Overweight OR "weight excess"):ti,ab,kw)<br>**AND**<br>([mh "Drug Therapy"] OR [mh "Weight Loss"] OR [mh "Weight Reduction Programs"] OR [mh "Bariatric Surgery"] OR [mh Liraglutide] OR [mh "Glucagon-Like Peptide-1 Receptor Agonists"] OR [mh sibutramine] OR [mh Topiramate] OR [mh Orlistat] OR [mh "Diet Therapy"] OR ((Drug NEXT Therap*) OR Pharmacotherap* OR (Weight NEXT Loss*) OR (Weight NEXT Reduction*) OR (Bariatric NEXT Surg*) OR (Metabolic NEXT Surger*) OR "Stomach Stapling" OR Liraglutide OR Victoza OR Saxenda OR "NN 2211" OR NN2211 OR semaglutide OR Rybelsus OR Wegovy OR Ozempic OR Tirzepatide OR "Glucagon-Like Peptide-1 Receptor Agonists" OR sibutramine OR "di-desmethylsibutramine" OR didesmethylsibutramine OR Reductil OR "mono-desmethylsibutramine" OR Meridia OR Phentermine OR Topiramate OR Topamax OR Orlistat OR Tetrahydrolipstatin OR THLP OR Tetrahydrolipastatin OR Xenical OR "bupropion hydrochloride, naltrexone hydrochoride drug combination" OR Mysimba OR Contrave OR (Diet NEXT Therap*) OR (Dietary NEXT Restriction*) OR (Dietary NEXT Modification*) OR (Diet NEXT Modification*)):ti,ab,kw)<br>**AND**<br>([mh "Bile Acids and Salts"] OR [mh "Fatty Acids, Volatile"] OR [mh Propionates] OR [mh Butyrates] OR [mh Acetates] OR [mh Indoles] OR [mh Indican] OR [mh Lipopolysaccharides] OR (trimethyloxamine OR "trimethylammonium oxide" OR "trimethylamine N-oxide" OR TMAO OR "trimethylamine oxide" OR (Bile NEXT Acid*) OR (Bile NEXT Salt*) OR ("Volatile Fatty" NEXT Acid*) OR ("Short Chain Fatty" NEXT Acid*) OR Propionate* OR Propanoate* OR (Propionic NEXT Acid*) OR (Propanoic NEXT Acid*) OR Butyrate* OR "Butyric Acids" OR "Butanoic Acids" OR Acetate* OR (Acetic NEXT Acid*) OR Indol* OR Indican OR "Indoxyl Sulfate" OR Glucosyltransferase* OR "IAGlc synthase" OR "IAA-glucose synthetase" OR "TI-3-P" OR Lipopolysaccharide* OR Lipoglycans):ti,ab,kw)

Table S1. Characteristics of Included Studies

Study		Intervention			Population			Outcomes	
**Identification**	**Design**	**Type**	**Description**	**Follow up**	**Sex (female, %)**	**Baseline BMI, kg/m²**	**n**	**Gut-microbiota metabolite**	**Δ BMI, %***
**Ahlin, 2021 [1]**	NRSI	Surgical	RYGB or BPD	~24 w	33.3	RYGB: 45.3 (5.7)<br>BPD: 55.9 (9.7)	15<br>(RYGB: 6; BPD: 9)	Indole derivative (IAA)	-26.5
**Ahmad, 2023 [2]**	NRSI	Surgical	SG	24 w	64.7	41.4 (9.7)	17	SCFAs (acetate, propionate, butyrate)	-24.6
**Albaugh, 2015 [3]**	NRSI	Surgical	RYGB	2 y	NR	47.4 (8.5)	21	Secondary BAs (DCA, UDCA)	-29.7
**Alkhatib, 2020 [4]**	RCT	Exercise / Supplement	HIIT program (3 sessions/wk) + anhydrous caffeine supplementation (HIIT+C) or placebo (HIIT+P)	8 w	100	HIIT+C: 26.7 (4.9)<br>HIIT+P: 27.4 (5.2)	24<br>(HIIT+ C: 12; HIIT+P: 12)	LPS	0.0
**Angelidi, 2023 [5]**	NRSI	Surgical	RYGB or SG	12 w	78.6	RYGB: 47.2 (7.3)<br>SG: 50.0 (7.5)	14<br>(RYGB: 6; SG: 8)	TMAO	-18.4
	RCT	Pharmacological	Liraglutide (daily dose; titrated +0.6 mg/week, up to 3.0 mg) or placebo	5 w	45.0	33.4 (2.9)	20	TMAO	-2.1
**Battillo, 2023 [6]**	RCT	Diet / Exercise	Low-calorie diet plus interval exercise (LCD+INT) or low-calorie diet (LCD) program alone	2 w	100	LCD+INT: 38.0 (7.6)<br>LCD: 37.8 (5.5)	23<br>(LCD+INT: 11; LCD: 12)	TMAO	-1.6
**Belgaumkar, 2016 [7]**	NRSI	Surgical	SG	24 w	66.7	60.6 (55.3; 65.8)	18	Secondary BAs (LCA, DCA, UDCA)	-23.6
**Braga, 2021 [8]**	NRSI	Diet, exercise and pharmacological	Medical care (MC): dietary and physical activity counseling, as well as anti-obesity medication when necessary	12 w	80.0	42.9 (4.7)	20	LPS	-4.2
	NRSI	Surgical	RYGB	12 w	80.0	42.0 (5.3)	15	LPS	-17.6
**Canyelles, 2022 [9]**	NRSI	Surgical	RYGB or SG	1 y	57.9	42.4 (39.9; 49.1)	19	TMAO	-28.7
**Chen, 2019 [10]**	NRSI	Surgical	RYGB or SG	12 w	84.2	RYGB: 43.0 (4.7)<br>SG: 43.4 (6.1)	19<br>(RYGB: 8; SG: 11)	Secondary BAs (total)	-14.3
**Clemente-Postigo, 2015 [11]**	NRSI	Surgical	SG or BPD; participants were classified as normoglycemic (NG) or with prediabetes/diabetes (P/D)	12 w	NR	BPD NG: 51.4 (3.6)<br>BPD P/D: 52.5 (5.1)<br>SG NG: 49.4 (5.9)<br>SG P/D: 48.6 (4.0)	50<br>(BPD NG: 9; BPD P/D: 17; SG NG: 11; SG P/D: 13)	LPS	-17.7

**Diao, 2024** [12]	RCT	Diet	Low-calorie DASH diet (LCDASH) or Low-Calorie diet (LCD); both involved daily energy restriction (−500 kcal/d) kcal less than total energy requirements	12 w	LCD: 63.0<br>LCDASH: 65.0	LCD: 27.7 (5.1)<br>LCDASH: 28.1 (6.5)	80<br>(LCD: 40; LCDASH: 40)	LPS, TMAO	-4.3
**Dutia, 2015** [13]	NRSI	Surgical	RYGB	2 y	100	43.3 (4.9)	13	Secondary BAs (total, LCA, DCA, UDCA)	-30.6
**Erickson, 2019** [14]	RCT	Diet / Exercise	Exercise (5 days/week, 80–85% HRmax) + hypocaloric diet (−500 kcal/day) (E+HYPO) or exercise + eucaloric diet (E+EU)	12 w	68.8	E+HYPO: 35.0 (5.0)<br>E+EU: 37.0 (5.0)	16<br>(E+ HYPO: 7; E+EU: 9)	TMAO	-6.8
**Escalona, 2016** [15]	NRSI	Surgical	SG	1 y	79.0	35.8 (3.5)	19	Secondary BAs (total, LCA, DCA, UDCA)	-30.7
**Fiamoncini, 2018** [16]	NRSI	Surgical	RYGB	12 w	64.1	44.1 (3.6)	39	Secondary BAs (DCA)	-20.9
**Guevara Cruz, 2024** [17]	RCT	Diet	Three hypocaloric diets (-500 kcal/d): ketogenic (KD), intermittent fasting (IF), or calorie restriction (CR), vs. habitual ad libitum diet. After 1 month, all groups received rifaximin; only pre-antibiotic data included.	4w	82.0	34.8 (4.20)	33	LPS	-3.3
**Guo, 2021** [18]	RCT	Diet	2 fasting dietary schedule (75% of energy restriction for two nonconsecutive days a week and an ad libitum diet the other five days) or routine diet without dietary instructions	8 w	46.1	28.0 (8.3)	21	LPS, TMAO	-3.6
**Han, 2022** [19]	NRSI	Surgical	RYGB or SG	1 y	71.0	37.8 (13.7)	38	TMAO, Secondary BA (LCA, DCA, UDCA)	-28.6
**Happonen, 2024** [20]	NRSI	Surgical	RYGB	24 w	70.0	44.5 (5.7)	30	LPS	-22.0
**Iversen, 2022** [21]	RCT	Diet	2-week run-in period with refined wheat products, followed by randomization to consume either refined wheat (W) or high-fiber rye (R) products. All participants received dietary guidance to maintain a 500 kcal/day energy deficit.	12 w	59.4	R: 29.8 (2.5)<br>W: 30.3 (2.5)	207<br>(R: 108; W: 99)	SCFAs (acetate, propionate, butyrate)	-2.5
**Jahansouz, 2016** [22]	RCT	Diet	Hypocaloric diet or RYGB (post-RYGB data pooled with the SG cohort, below)	1 w	71.4	43.3 (5.6)	14	Secondary BAs (DCA, UDCA)	-3.23
	NRSI	Surgical	RYGB or SG	1 w	68.4	RYGB: 43.0 (5.9)<br>SG: 45.8 (8.8)	25<br>(RYGB: 13; SG: 12)	Secondary BAs (DCA, UDCA)	-2.9

**Jianguo, 2021** [23]	RCT	Diet / Supplement	Group A: Microecological preparation (glutamine, Bifidobacterium trifecta, probiotics, probiotic metabolites) + improved low-carbon diet (1023-1093 kcal/day). Group B: Improved low-carbon diet only (1023-1093 kcal/day.); Groups C and D: Balanced diet (individuals with obesity and healthy controls - arms not included).	24 w	49.0	A: 31.0 (5.2)<br>B: 30.1 (3.6)	50<br>(A: 25; B: 25)	LPS	-11.8
**Jørgensen, 2015** [24]	NRSI	Surgical	RYGB, divided in two groups: type 2 diabetes (T2D) or normal glucose tolerance (NTG)	1 y	60.0	T2D: 43.1 (5.1)<br>NTG: 41.5 (4.8)	25<br>(T2D: 13; NTG: 12)	Secondary BAs (total)	-23.2
**Kindel, 2018** [25]	NRSI	Surgical	SG	12 w	82.0	45.0 (6.8)	28	Secondary BAs (LCA, DCA)	-13.7
**Kohli, 2013** [26]	NRSI	Surgical	LAGB or RYGB	until 20% weight loss	83.3	NR	18<br>(LAGB: 10; RYGB: 8)	Secondary BAs (total)	>-20.0
**Kokkinos, 2024** [27]	NRSI	Surgical	RYGB or SG	10 y	82.1	49.6 (6.8)	28	TMAO	-25.1
**Kwon, 2021** [28]	NRSI	Surgical	Laparoscopic SG	12 w	73.9	39.3 (5.3)	23	Indole derivative (ILA, IPA, IAA)	-19.0
**Lalloyer, 2023** [29]	NRSI	Surgical	RYGB	1y	26.9	42.0 (5.0)	26	Secondary BAs (total, LCA, DCA, UDCA)	-28.6
**Lee, 2021** [30]	RCT	Surgical	Laparoscopic RYGB or SG	24 w	29.0	RYGB: 39.2 (5.9)<br>SG: 38.9 (6.5)	38<br>(RYGB: 17; SG: 21)	TMAO	-27.6
**Li, 2019** [31]	NRSI	Surgical	Laparoscopic SG	4 w	83.3	39.4 (8.2)	30	LPS	-14.7
**Linsalata, 2023** [32]	NRSI	Diet	Very Low Calorie Ketogenic Diet (450-800 kcal/day)	8 w	54.0	35.6 (1.1)	24	LPS	-11.2
**Machado, 2020** [33]	NRSI	Surgical	RYGB, with cholecystectomy (WC) or without cholecystectomy (NC)	12 w	100	WC: 47.0 (4.6)<br>NC: 44.4 (6.0)	28<br>(WC: 7; NC: 21)	Secondary BAs (LCA, DCA)	-3.0
**Malin, 2024** [34]	RCT	Exercise	12 supervised, work-matched 60-minute cycle ergometer sessions over 13 days, with a rest day on day 8. The CONT group exercised continuously at 70% HRpeak, while the INT group alternated between 3 minutes at 90% HRpeak and 50% HRpeak during each session	2w	82.6	INT: 32.1 (4.0)<br>CONT: 34.0 (5.9)	23<br>(INT: 11; CONT: 12)	Secondary BAs (LCA, DCA, UDCA)	-0.5
**Martínez-Sánchez, 2023** [35]	NRSI	Surgical	RYGB	1 y	80.0	43.6 (5.2)	51	SCFAs (acetate, propionate, butyrate)	-34.0

**Mercer, 2021**[36]	NRSI	Diet / Exercise	Calorie-restricted diet for a 10% body mass loss over 14 weeks (500-600 kcal/day reduction). Exercise started at 30 minutes, 4 days/week at 60-70% maximal HR (weeks 0-4), then increased to 40 minutes (weeks 5-8), and intensity rose to 75% maximal HR from week 9 onward.	14 w	100	33.9 (2.4)	11	Secondary BAs (DCA, UDCA)	-5.6
**Mika, 2018** [37]	NRSI	Surgical	SG, RYGB or OLGB	12 w	100	LSG: 41.5 (4.2)<br>RYGB: 40.6 (3.2)<br>OLGB: 44.3 (3.3)	26<br>(LSG: 7; RYGB: 9; OLGB: 10)	Secondary BAs (total)	-21.0
**Monte, 2012** [38]	NRSI	Surgical	RYGB	24 w	73.3	52.1 (13.0)	15	LPS	-22.5
**Nakatani, 2009** [39]	NRSI	Surgical	malabsorptive procedures (MP), being them RYGB or SG+duodenal jejunal bypass surgery; or restrictive procedures (RP), being them SG or LAGB	12 w	53.0	MP: 43.0 (6.5)<br>RP: 45.3 (11.2)	34<br>(MP:19; RP: 15)	Secondary BAs (total)	-16.6
**Nemati, 2018** [40]	RCT	Surgical	SG or RYGB	1 y	51.0	SG: 40.0 (6.6)<br>RYGB: 40.0 (7.0)	61<br>(SG: 29; RYGB: 32)	Secondary BAs (total, LCA, DCA)	-25.1
**Netto Cândido, 2021** [41]	RCT	Diet	3 tested oil types (SO = soybean oil, EVO = extra virgin olive, CO = coconut oil), as part of an energy-restricted (-500 kcal/day)	9 w	100	SO: 30.1 (5.8)<br>EVO: 29.7 (5.3)<br>CO: 30.0 (6.0)	52<br>(SO: 17; EVO: 19; CO: 16)	LPS	-1.6
**Nikard, 2023** [42]	RCT	Diet/Supplement	Low calorie diet (-500kcal) + 5 g/day thylakoid-rich spinach extract (LCD+SE) or low-calorie diet + placebo (LCD+P)	12 w	100	LCD+SE: 35.1 (2.2)<br>LCD+P: 35.3 (2.8)	44<br>(LCD+SE: 21; LCD+P: 23)	LPS	-5.6
**Palmiotti, 2024** [43]	NRSI	Diet / Exercise	Advice on healthy eating, exercise and behavioral therapy; 18 sessions with a dietitian and psychologist, including 1.5 h of exercise under the supervision of a physical therapist	75 w	69.0	39.7 (5.4)	45	Secondary BAs (total)	-6.5
**Risstad, 2017** [44]	RCT	Surgical	RYGB or BPD/DS	5 y	RYGB: 74.2<br>BPD/DS: 65.5	RYGB: 54.8 (3.2)<br>BPD/DS: 55.2 (3.5)	60<br>(RYGB: 31; BPD/DS: 29)	Secondary BAs (total, LCA, DCA, UDCA)	-30.2
**Rooholahzadegan, 2023** [45]	RCT	Diet	Calorie-restricted DASH diet, -500 kcal/day, following DASH pattern (CR-DASH) or Calorie-restricted control diet, -500 kcal/day (CR-C)	8 w	CR-DASH: 65.0<br>CR-C: 60.0	CR-DASH: 33.4 (4.1)<br>CR-C: 34.0 (3.6)	40<br>(CR-DASH: 20; CR-C: 20)	LPS	-7.2
**Sachdev, 2016** [46]	RCT	Surgical	RYGB or	1 y	53.0	36.2 (2.7)	15	Secondary BAs (total, LCA, DCA, UDCA)	-28.9
		Non-Surgical	Intensive lifestyle and medical management	1y	46.7	35.5 (3.5)	15	Secondary BAs (total, LCA, DCA, UDCA)	-11.3

**Scheithauer, 2022** [47]	NRSI	Surgical	RYGB; analysis was categorized between people without diabetes (ND) and with diabetes (T2D)	1 y	ND: 54.0 T2D: 60.0	40.7 (4.8)	40 (ND: 20; T2D: 20)	LPS	-27.3
**Shen, 2019** [48]	NRSI	Surgical	RYGB or SG; study grouped two cohorts, New York (NY) and Barcelona (BCN)	1 y	BCN: 83.3 NY: 85.7	46.1 (6.3)	26 (RYGB: 19; SG: 7)	TMAO, SCFA (acetate, propionate, butyrate)	-33.5
**Shi, 2021** [49]	NRSI	Surgical	RYGB	24 w	57.0	37.7 (6.7)	14	TMAO	-26.8
**Simonen, 2012** [50]	NRSI	Surgical	RYGB	1 y	90.0	46.1 (5.9)	30	Secondary BAs (total, LCA, DCA, UDCA)	-25.6
**Spinelli, 2016** [51]	NRSI	Surgical	RYGB	1 y	86.0	42.9 (2.7)	7	Secondary BAs (DCA, UDCA)	-37.1
**Straniero, 2017** [52]	NRSI	Diet	Low-calorie diet (800-1100 kcal/day)	4 w	100	41.7 (2.6)	10	Secondary BAs (LCA, DCA, UDCA)	NR
**Sun, 2020** [53]	NRSI	Diet	Fasting therapy intervention, including 1 day of moderate calorie restriction (<800 kcal), 5 days of intense restriction (200 kcal), and 1 day of gradual reintroduction (800–1000 kcal). Participants received bowel-cleansing powder, 2L of water, a 200 kcal liquid meal, vegetable soup, and L-carnitine (2g BID). All participants were hospitalized during the intervention.	1 w	62.0	29.9 (2.8)	13	TMAO	-5.3
**Thöni, 2017** [54]	NRSI	Surgical	LAGB	1 y	65.0	43.5 (4.0)	20	Secondary BAs (total, LCA, DCA, UDCA)	-17.7
**Tilves, 2022** [55]	RCT	Diet / Exercise	Coach-directed weight loss (a remotely delivered lifestyle intervention consisting of increased physical activity, reduced calorie intake, and guidance to eat the Dietary Approaches to Stopping Hypertension (DASH) diet); compared to metformin or self-directed weight loss (arms not included).	1 y	78.4	34.9 (5.4)	29	SCFAs (acetate, propionate, butyrate)	-3.5
**Trøseid, 2013** [56]	NRSI	Surgical	Lifestyle changes resulting in a minimum of 10% weight loss preoperatively, followed by laparoscopic RYGB (if BMI <50kg/m2) or DS (if BMI >50kg/m2)	1 y	69.0	45.1 (7.3)	49	LPS	NR
**Trøseid, 2016** [57]	NRSI	Surgical	Lifestyle changes resulting in a minimum of 10% weight loss preoperatively, followed by laparoscopic RYGB (if BMI <50kg/m2) or DS (if BMI >50kg/m2)	1 y	56.0	44.8 (6.9)	34	TMAO	NR
**van Nierop, 2017** [58]	NRSI	Diet	450 kcal/day diet for 14 days; The diet consisted of 3 sachets of a liquid meal replacement formula	2 w	33.0	39.8 (6.9)	12	Secondary BAs (DCA)	-3.5

**van Nierop, 2019 [59]**	NRSI	Surgical	Duodenal-Jejunal Bypass Liner	24 w	18.0	37.0 (5.4)	17	Secondary BAs (DCA, UDCA)	-11.1
**Verde, 2024a [60]**	NRSI	Diet	VLCKD active phase: total energy intake <800 kcal/day. This energy was derived from a distribution of 13% from carbohydrates (less than 30 g per day), 43% from protein (1.3 g per kilogram of ideal body weight), and 44% from fat.	4 w	100	36.0 (5.9)	12	TMAO	-7.9
**Verde, 2024b [61]**	NRSI	Diet	VLCKD active phase: total energy intake <800 kcal/day. This energy was derived from a distribution of 13% from carbohydrates (less than 30 g per day), 43% from protein (1.3 g per kilogram of ideal body weight), and 44% from fat.	45 d	100	32.7 (1.3)	31	TMAO	-8.1
**Werling, 2013 [62]**	NRSI	Surgical	RYGB	15 m	68.0	43.7 (39.3; 49.2)	63	Secondary BAs (DCA, UDCA)	-33.2
**Wijayatunga, 2018 [63]**	NRSI	Surgical	RYGB	24 w	75.0	46.8 (6.2)	20	SCFAs (acetate, butyrate)	-26.7
**Yang, 2021 [64]**	NRSI	Surgical	RYGB or SG	12-24 w	RYGB: 95.0<br>SG: 81.0	RYGB: 46.4 (4.7)<br>SG: 56.3 (7.7)	42<br>(RYGB: 21; SG: 21)	Secondary BAs (DCA)	NR
**Yang, 2023 [65]**	RCT	Diet	Hypocaloric diet (-500 kcal/day), including 1.5 oz mixed tree nuts (MTN) or pretzels (PTZ) for 12 weeks; isocaloric diet for another 12 weeks of weight maintenance	24 w	MTN: 69.6<br>PTZ: 84.0	MTN: 31.1 (2.6)<br>PTZ: 30.7 (2.4)	95<br>(MTN: 56; PTZ: 39)	Indole derivative (IPA, IAA)	NR
**Yao, 2021 [66]**	NRSI	Surgical	Laparoscopic SG	24 w	50.0	39.7 (3.7)	14	Secondary BAs (LCA, DCA, UDCA)	-20.7
**Zhang, 2024 [67]**	NRSI	Diet / Exercise	High-protein diet and moderate exercise training, in addition to conventional medication	1 y	58.3	36.7 (5.9)	12	SCFAs (acetate, propionate, butyrate)	-19.3

Data presented as mean (SD) or median (IQR)

* For studies with multiple intervention arms included in the meta-analysis, Δ BMI was calculated using the weighted average across arms, when available. If not provided, a pooled mean change was computed from the combined pre- and post-intervention BMI values

Legend | BMI = Body Mass Index; BAs = bile acids; LPS = lipopolysaccharide; TMAO = trimethylamine N-oxide; SCFAs = short-chain fatty acids; LCA = lithocholic acid; DCA = deoxycholic acid; UDCA = ursodeoxycholic acid; IPA = indole-3-propionic acid; ILA = indole-3-lactic acid; IAA = indole-3-acetic acid; RYGB = Roux-en-Y Gastric Bypass; SG = sleeve gastrectomy; BPD = biliopancreatic diversion; DS = duodenal switch; OLGB = omega-loop gastric bypass; LAGB = laparoscopic adjustable gastric banding; HIIT = high-intensity interval training; VLCKD = Very low-calorie ketogenic diet | RCT = randomized controlled trial; NRSI = non-randomized study of intervention; SD = standard deviation; IQR = interquartile range (p25; p75); NR = not reported

1. Ahlin S, Cefalo C, Bondia-Pons I, et al. Metabolite Changes After Metabolic Surgery – Associations to Parameters Reflecting Glucose Homeostasis and Lipid Levels. *Front Endocrinol*. 2021;12((Ahlin S., sofie.ahlin@gu.se) Department of Molecular and Clinical Medicine, Institute of Medicine, The Sahlgrenska Academy at the University of Gothenburg, Gothenburg, Sweden). doi:10.3389/fendo.2021.786952

2. Ahmad AF, Caparrós-Martín JA, Lee S, et al. Gut Microbiome and Associated Metabolites Following Bariatric Surgery and Comparison to Healthy Controls. *Microorganisms*. 2023;11(5). doi:10.3390/microorganisms11051126

3. Albaugh V, Flynn C, Cai S, Xiao Y, Tamboli R, Abumrad N. Early Increases in Bile Acids Post Roux-en-Y Gastric Bypass Are Driven by Insulin-Sensitizing, Secondary Bile Acids. *J Clin Endocrinol Metab*. 2015;100(9):E1225-33. doi:10.1210/jc.2015-2467

4. Alkhatib A, Hsieh M, Kuo C, Hou C. Caffeine Optimizes HIIT Benefits on Obesity-associated Metabolic Adversity in Women. *Med Sci Sports Exerc*. 2020;52(8):1793-1800. doi:10.1249/MSS.0000000000002311

5. Angelidi AM, Kokkinos A, Sanoudou D, et al. Early metabolomic, lipid and lipoprotein changes in response to medical and surgical therapeutic approaches to obesity. *Metabolism*. 2023;138:155346. doi:10.1016/j.metabol.2022.155346

6. Battillo DJ, Malin SK. Impact of Caloric Restriction and Exercise on Trimethylamine N-Oxide Metabolism in Women with Obesity. *Nutrients*. 2023;15(6). doi:10.3390/nu15061455

7. Belgaumkar AP, Vincent RP, Carswell KA, et al. Changes in Bile Acid Profile After Laparoscopic Sleeve Gastrectomy are Associated with Improvements in Metabolic Profile and Fatty Liver Disease. *Obes Surg*. 2016;26(6):1195-1202. doi:10.1007/s11695-015-1878-1

8. Braga TG, Graças Coelho de Souza MD, Menezes M, et al. Dipeptidyl peptidase-4 activity, lipopolysaccharide, C-reactive protein, glucose metabolism, and gut peptides 3 months after bariatric surgery. *Surg Obes Relat Dis Off J Am Soc Bariatr Surg*. 2021;17(1):113-120. doi:10.1016/j.soard.2020.08.030

9. Canyelles M, Pérez A, Junza A, et al. Divergent Effects of Glycemic Control and Bariatric Surgery on Circulating Concentrations of TMAO in Newly Diagnosed T2D Patients and Morbidly Obese. *Diagnostics*. 2022;12(11). doi:10.3390/diagnostics12112783

10. Chen Y, Lu J, Nemati R, Plank LD, Murphy R. Acute Changes of Bile Acids and FGF19 After Sleeve Gastrectomy and Roux-en-Y Gastric Bypass. *Obes Surg*. 2019;29(11):3605-3621. doi:10.1007/s11695-019-04040-x

11. Clemente-Postigo M, Roca-Rodriguez M del M, Camargo A, Ocaña-Wilhelmi L, Cardona F, Tinahones FJ. Lipopolysaccharide and lipopolysaccharide-binding protein levels and their relationship to early metabolic improvement after bariatric surgery. *Surg Obes Relat Dis Off J Am Soc Bariatr Surg*. 2015;11(4):933-939. doi:10.1016/j.soard.2014.11.030

12. Diao Z, Molludi J, Latef Fateh H, Moradi S. Comparison of the low-calorie DASH diet and a low-calorie diet on serum TMAO concentrations and gut microbiota composition of adults with overweight/obesity: a randomized control trial. *Int J Food Sci Nutr*. 2024;75(2):207-220. doi:10.1080/09637486.2023.2294685

13. Dutia R, Embrey M, O'Brien CS, et al. Temporal changes in bile acid levels and 12α-hydroxylation after Roux-en-Y gastric bypass surgery in type 2 diabetes. *Int J Obes 2005*. 2015;39(5):806-813. doi:10.1038/ijo.2015.1

14. Erickson M, Malin S, Wang Z, Brown J, Hazen S, Kirwan J. Effects of Lifestyle Intervention on Plasma Trimethylamine N-Oxide in Obese Adults. *Nutrients*. 2019;11(1). doi:10.3390/nu11010179

15. Escalona A, Muñoz R, Irribarra V, Solari S, Allende F, Francisco Miquel J. Bile acids synthesis decreases after laparoscopic sleeve gastrectomy. *Surg Obes Relat Dis Off J Am Soc Bariatr Surg*. 2016;12(4):763-769. doi:10.1016/j.soard.2015.11.015

16. Fiamoncini J, Barbosa CF, Arnoni Junior JR, et al. Roux-en-y gastric bypass surgery induces distinct but frequently transient effects on acylcarnitine, bile acid and phospholipid levels. *Metabolites*. 2018;8(4). doi:10.3390/metabo8040083

17. Guevara-Cruz M, Hernández-Gómez KG, Condado-Huerta C, et al. Intermittent fasting, calorie restriction, and a ketogenic diet improve mitochondrial function by reducing lipopolysaccharide signaling in monocytes during obesity: A randomized clinical trial. *Clin Nutr*. 2024;43(8):1914-1928. doi:10.1016/j.clnu.2024.06.036

18. Guo Y, Luo S, Ye Y, Yin S, Fan J, Xia M. Intermittent Fasting Improves Cardiometabolic Risk Factors and Alters Gut Microbiota in Metabolic Syndrome Patients. *J Clin Endocrinol Metab*. 2021;106(1):64-79. doi:10.1210/clinem/dgaa644

19. Han Y, Kim G, Ahn E, et al. Integrated metagenomics and metabolomics analysis illustrates the systemic impact of the gut microbiota on host metabolism after bariatric surgery. *Diabetes Obes Metab*. 2022;24(7):1224-1234. doi:10.1111/dom.14689

20. Happonen N, Härma MA, Akhi R, et al. Impact of RYGB surgery on plasma immunoglobulins: association between blood pressure and glucose levels six months after surgery. *APMIS Acta Pathol Microbiol Immunol Scand*. 2024;132(3):187-197. doi:10.1111/apm.13366

21. Iversen KN, Dicksved J, Zoki C, et al. The Effects of High Fiber Rye, Compared to Refined Wheat, on Gut Microbiota Composition, Plasma Short Chain Fatty Acids, and Implications for Weight Loss and Metabolic Risk Factors (the RyeWeight Study). *Nutrients*. 2022;14(8). doi:10.3390/nu14081669

22. Jahansouz C, Xu H, Hertzel AV, et al. Bile Acids Increase Independently From Hypocaloric Restriction After Bariatric Surgery. *Ann Surg*. 2016;264(6):1022-1028. doi:10.1097/SLA.0000000000001552

23. Jianguo L, Yong P, Hanyu L, et al. Effect of microecological preparation combined with an improved low-carbon diet on fat metabolism and intestinal barrier function in obese patients. *Chin J Tissue Eng Res*. 2021;25(29):4672-4679. doi:10.12307/2021.167

24. Jørgensen NB, Dirksen C, Bojsen-Møller KN, et al. Improvements in glucose metabolism early after gastric bypass surgery are not explained by increases in total bile acids and fibroblast growth factor 19 concentrations. *J Clin Endocrinol Metab*. 2015;100(3):E396-406. doi:10.1210/jc.2014-1658

25. Kindel TL, Krause C, Helm MC, et al. Increased glycine-amidated hyocholic acid correlates to improved early weight loss after sleeve gastrectomy. *Surg Endosc*. 2018;32(2):805-812. doi:10.1007/s00464-017-5747-y

26. Kohli R, Bradley D, Setchell KD, Eagon JC, Abumrad N, Klein S. Weight loss induced by Roux-en-Y gastric bypass but not laparoscopic adjustable gastric banding increases circulating bile acids. *J Clin Endocrinol Metab*. 2013;98(4):E708-712. doi:10.1210/jc.2012-3736

27. Kokkinos A, Tsilingiris D, Simati S, et al. Bariatric surgery, through beneficial effects on underlying mechanisms, improves cardiorenal and liver metabolic risk over an average of ten years of observation: A longitudinal and a case-control study. *Metabolism*. 2024;152:155773. doi:10.1016/j.metabol.2023.155773

28. Kwon Y, Jang M, Lee Y, Ha J, Park S. Metabolomic Analysis of the Improvements in Insulin Secretion and Resistance After Sleeve Gastrectomy: Implications of the Novel Biomarkers. *Obes Surg*. 2021;31(1):43-52. doi:10.1007/s11695-020-04925-2

29. Lalloyer F, Mogilenko DA, Verrijken A, et al. Roux-en-Y gastric bypass induces hepatic transcriptomic signatures and plasma metabolite changes indicative of improved cholesterol homeostasis. *J Hepatol*. 2023;79(4):898-909. doi:10.1016/j.jhep.2023.05.012

30. Lee SJ, Park YS, Kim YJ, et al. Changes in Trimethylamine-N-oxide Levels in Obese Patients following Laparoscopic Roux-en-Y Gastric Bypass or Sleeve Gastrectomy in a Korean Obesity Surgical Treatment Study (KOBESS). *J Clin Med*. 2021;10(21). doi:10.3390/jcm10215091

31. Li Y, Guan W, Ma S, et al. Lipopolysaccharide and inflammatory cytokines levels decreased after sleeve gastrectomy in Chinese adults with obesity. *Endocr J*. 2019;66(4):337-347. doi:10.1507/endocrj.EJ18-0446

32. Linsalata M, Russo F, Riezzo G, et al. The Effects of a Very-Low-Calorie Ketogenic Diet on the Intestinal Barrier Integrity and Function in Patients with Obesity: A Pilot Study. *Nutrients*. 2023;15(11). doi:10.3390/nu15112561

33. Machado NM, Cardinelli C de S, Shen T, Santo MA, Torrinhas RS, Waitzberg DL. Cholecystectomy - a potential selection bias in studies assessing the metabolic effects of bariatric surgeries. *Sci Rep*. 2020;10(1):10683. doi:10.1038/s41598-020-66688-1

34. Malin SK, Syeda USA. Exercise Training Independent of Intensity Lowers Plasma Bile Acids in Prediabetes. *Med Sci Sports Exerc*. 2024;56(6):1009-1017. doi:10.1249/MSS.0000000000003384

35. Martínez-Sánchez MA, Balaguer-Román A, Fernández-Ruiz VE, et al. Plasma short-chain fatty acid changes after bariatric surgery in patients with severe obesity. *Surg Obes Relat Dis Off J Am Soc Bariatr Surg*. 2023;19(7):727-734. doi:10.1016/j.soard.2022.12.041

36. Mercer KE, Maurer A, Pack LM, et al. Exercise training and diet-induced weight loss increase markers of hepatic bile acid (BA) synthesis and reduce serum total BA concentrations in obese women. *Am J Physiol Endocrinol Metab*. 2021;320(5):E864-E873. doi:10.1152/ajpendo.00644.2020

37. Mika A, Kaska L, Proczko-Stepaniak M, et al. Evidence That the Length of Bile Loop Determines Serum Bile Acid Concentration and Glycemic Control After Bariatric Surgery. *Obes Surg*. 2018;28(11):3405-3414. doi:10.1007/s11695-018-3314-9

38. Monte SV, Caruana JA, Ghanim H, et al. Reduction in endotoxemia, oxidative and inflammatory stress, and insulin resistance after Roux-en-Y gastric bypass surgery in patients with morbid obesity and type 2 diabetes mellitus. *Surgery*. 2012;151(4):587-593. doi:10.1016/j.surg.2011.09.038

39. Nakatani H, Kasama K, Oshiro T, Watanabe M, Hirose H, Itoh H. Serum bile acid along with plasma incretins and serum high-molecular weight adiponectin levels are increased after bariatric surgery. *Metabolism*. 2009;58(10):1400-1407. doi:10.1016/j.metabol.2009.05.006

40. Nemati R, Lu J, Dokpuang D, Booth M, Plank LD, Murphy R. Increased Bile Acids and FGF19 After Sleeve Gastrectomy and Roux-en-Y Gastric Bypass Correlate with Improvement in Type 2 Diabetes in a Randomized Trial. *Obes Surg*. 2018;28(9):2672-2686. doi:10.1007/s11695-018-3216-x

41. Netto Cândido T, da Silva L, Cândido F, et al. Effect of the ingestion of vegetable oils associated with energy-restricted normofat diet on intestinal microbiota and permeability in overweight women. *Food Res Int Ott Ont*. 2021;139:109951. doi:10.1016/j.foodres.2020.109951

42. Nikrad N, Farhangi MA, Fard Tabrizi FP, Vaezi M, Mahmoudpour A, Mesgari-Abbasi M. The effect of calorie-restriction along with thylakoid membranes of spinach on the gut-brain Axis Pathway and oxidative stress biomarkers in obese women with polycystic ovary syndrome: a Randomized, Double-blind, placebo-controlled clinical trial. *J Ovarian Res*. 2023;16(1):216. doi:10.1186/s13048-023-01288-x

43. Palmiotti A, Berk KA, Koehorst M, et al. Reversal of insulin resistance in people with obesity by lifestyle-induced weight loss does not impact the proportion of circulating 12α-hydroxylated bile acids. *Diabetes Obes Metab*. Published online July 3, 2024. doi:10.1111/dom.15754

44. Risstad H, Kristinsson JA, Fagerland MW, et al. Bile acid profiles over 5 years after gastric bypass and duodenal switch: results from a randomized clinical trial. *Surg Obes Relat Dis Off J Am Soc Bariatr Surg*. 2017;13(9):1544-1553. doi:10.1016/j.soard.2017.05.024

45. Rooholahzadegan F, Arefhosseini S, Tutunchi H, Badali T, Khoshbaten M, Ebrahimi-Mameghani M. The effect of DASH diet on glycemic response, meta-inflammation and serum LPS in obese patients with NAFLD: a double-blind controlled randomized clinical trial. *Nutr Metab*. 2023;20(1). doi:10.1186/s12986-023-00733-4

46. Sachdev S, Wang Q, Billington C, et al. FGF 19 and Bile Acids Increase Following Roux-en-Y Gastric Bypass but Not After Medical Management in Patients with Type 2 Diabetes. *Obes Surg*. 2016;26(5):957-965. doi:10.1007/s11695-015-1834-0

47. Scheithauer TPM, Davids M, Winkelmeijer M, et al. Compensatory intestinal antibody response against pro-inflammatory microbiota after bariatric surgery. *Gut Microbes*. 2022;14(1):2031696. doi:10.1080/19490976.2022.2031696

48. Shen N, Caixàs A, Ahlers M, et al. Longitudinal changes of microbiome composition and microbial metabolomics after surgical weight loss in individuals with obesity. *Surg Obes Relat Dis Off J Am Soc Bariatr Surg*. 2019;15(8):1367-1373. doi:10.1016/j.soard.2019.05.038

49. Shi Q, Wang Q, Zhong H, et al. Roux-en-Y Gastric Bypass Improved Insulin Resistance via Alteration of the Human Gut Microbiome and Alleviation of Endotoxemia. *BioMed Res Int*. 2021;2021:5554991. doi:10.1155/2021/5554991

50. Simonen M, Dali-Youcef N, Kaminska D, et al. Conjugated bile acids associate with altered rates of glucose and lipid oxidation after Roux-en-Y gastric bypass. *Obes Surg*. 2012;22(9):1473-1480. doi:10.1007/s11695-012-0673-5

51. Spinelli V, Lalloyer F, Baud G, et al. Influence of Roux-en-Y gastric bypass on plasma bile acid profiles: a comparative study between rats, pigs and humans. *Int J Obes 2005*. 2016;40(8):1260-1267. doi:10.1038/ijo.2016.46

52. Straniero S, Rosqvist F, Edholm D, et al. Acute caloric restriction counteracts hepatic bile acid and cholesterol deficiency in morbid obesity. *J Intern Med*. 2017;281(5):507-517. doi:10.1111/joim.12599

53. Sun J, Zhang T, Zhang L, Ke B, Qin J. Fasting Therapy Contributes to the Improvement of Endothelial Function and Decline in Vascular Injury-Related Markers in Overweight and Obese Individuals via Activating Autophagy of Endothelial Progenitor Cells. *Evid Based Complement Alternat Med*. 2020;2020((Sun J., sunjp3@mail3.sysu.edu.cn; Qin J., himybox@yeah.net) Department of Traditional Chinese Medicine, First Affiliated Hospital, Sun Yat-Sen University, Guangzhou, Guangdong, China). doi:10.1155/2020/3576030

54. Thöni V, Pfister A, Melmer A, et al. Dynamics of Bile Acid Profiles, GLP-1, and FGF19 After Laparoscopic Gastric Banding. *J Clin Endocrinol Metab*. 2017;102(8):2974-2984. doi:10.1210/jc.2017-00235

55. Tilves C, Yeh HC, Maruthur N, et al. Increases in Circulating and Fecal Butyrate are Associated With Reduced Blood Pressure and Hypertension: Results From the SPIRIT Trial. *J Am Heart Assoc*. 2022;11(13):e024763. doi:10.1161/JAHA.121.024763

56. Trøseid M, Nestvold TK, Rudi K, Thoresen H, Nielsen EW, Lappegård KT. Plasma lipopolysaccharide is closely associated with glycemic control and abdominal obesity: evidence from bariatric surgery. *Diabetes Care*. 2013;36(11):3627-3632. doi:10.2337/dc13-0451

57. Trøseid M, Hov JR, Nestvold TK, et al. Major Increase in Microbiota-Dependent Proatherogenic Metabolite TMAO One Year After Bariatric Surgery. *Metab Syndr Relat Disord*. 2016;14(4):197-201. doi:10.1089/met.2015.0120

58. van Nierop FS, Kulik W, Endert E, et al. Effects of acute dietary weight loss on postprandial plasma bile acid responses in obese insulin resistant subjects. *Clin Nutr Edinb Scotl*. 2017;36(6):1615-1620. doi:10.1016/j.clnu.2016.10.006

59. van Nierop FS, de Jonge C, Kulik W, et al. Duodenal-jejunal lining increases postprandial unconjugated bile acid responses and disrupts the bile acid-FXR-FGF19 axis in humans. *Metabolism*. 2019;93:25-32. doi:10.1016/j.metabol.2018.12.009

60. Verde L, Cacciapuoti S, Caiazzo G, et al. Very low-calorie ketogenic diet (VLCKD) in the management of hidradenitis suppurativa (Acne Inversa): an effective and safe tool for improvement of the clinical severity of disease. Results of a pilot study. *J Transl Med*. 2024;22(1):149. doi:10.1186/s12967-024-04853-0

61. Verde L, Frias-Toral E, Cacciapuoti S, et al. Very low-calorie ketogenic diet (VLCKD): a therapeutic nutritional tool for acne? *J Transl Med*. 2024;22(1):322. doi:10.1186/s12967-024-05119-5

62. Werling M, Vincent RP, Cross GF, et al. Enhanced fasting and post-prandial plasma bile acid responses after Roux-en-Y gastric bypass surgery. *Scand J Gastroenterol*. 2013;48(11):1257-1264. doi:10.3109/00365521.2013.833647

63. Wijayatunga NN, Sams VG, Dawson JA, Mancini ML, Mancini GJ, Moustaid-Moussa N. Roux-en-Y gastric bypass surgery alters serum metabolites and fatty acids in patients with morbid obesity. *Diabetes Metab Res Rev*. 2018;34(8):e3045. doi:10.1002/dmrr.3045

64. Yang C, Brecht J, Weiß C, et al. Serum Glucagon, Bile Acids, and FGF-19: Metabolic Behavior Patterns After Roux-en-Y Gastric Bypass and

Vertical Sleeve Gastrectomy. *Obes Surg*. 2021;31(11):4939-4946. doi:10.1007/s11695-021-05677-3

65. Yang J, Lee R, Schulz Z, et al. Mixed Nuts as Healthy Snacks: Effect on Tryptophan Metabolism and Cardiovascular Risk Factors. *Nutrients*. 2023;15(3). doi:10.3390/nu15030569

66. Yao J, Kovalik JP, Lai OF, et al. Effects of laparoscopic sleeve gastrectomy on concentration and composition of bile acids in an Asian population with morbid obesity. *Proc Singap Healthc*. 2021;30(2):111-116. doi:10.1177/2010105820952489

67. Zhang S, Zhang Y, Li J, et al. Butyrate and Propionate are Negatively Correlated with Obesity and Glucose Levels in Patients with Type 2 Diabetes and Obesity. *Diabetes Metab Syndr Obes*. 2024;17((Zhang S.; Li J.; Wang X.; Du M.; Li C., li_chunjun@126.com) Department of Endocrinology, Health Management Center, Tianjin Union Medical Center, Nankai University Affiliated Hospital, Tianjin, China):1533-1541. doi:10.2147/DMSO.S434499

Table S2. Excluded studies and reason for exclusion, after full-text analysis

First Author	Year	Title	Reason for exclusion
Abbasi, F	2008	Comparison of three treatment approaches to decreasing cardiovascular disease risk in nondiabetic insulin-resistant dyslipidemic subjects.	No assessment of outcome of interest
Ahmad, N	2013	Roux-en-Y gastric bypass normalizes the blunted postprandial bile acid excursion associated with obesity.	No assessment of outcome of interest
Argyridou, S	2021	Evaluation of an 8-Week Vegan Diet on Plasma Trimethylamine-N-Oxide and Postchallenge Glucose in Adults with Dysglycemia or Obesity.	No weight loss interventions
Benítez-Páez, A	2021	Sex, Food, and the Gut Microbiota: Disparate Response to Caloric Restriction Diet with Fiber Supplementation in Women and Men.	Non-extractable or insufficient data
Bini, S	2022	Differential effects of bariatric surgery on plasma levels of ANGPTL3 and ANGPTL4.	No assessment of outcome of interest
Binobead, M	2023	Effect of Low-Carbohydrate Diet on Beta-Hydroxybutyrate Ketogenesis Metabolic Stimulation and Regulation of NLRP3 Ubiquitination in Obese Saudi Women.	No assessment of outcome of interest
Bonakdar, R	2020	Detoxification Enhanced Lifestyle Intervention Targeting Endotoxemia (DELITE) in the Setting of Obesity and Pain: Results of a Pilot Group Intervention.	No assessment of outcome of interest
Calabrese, F	2024	Gut microbiota and fecal volatilome profile inspection in metabolically healthy and unhealthy obesity phenotypes.	No assessment of outcome of interest
Coleman, M	2005	Urinary ketones reflect serum ketone concentration but do not relate to weight loss in overweight premenopausal women following a low-carbohydrate/high-protein diet.	No assessment of outcome of interest
De Vuono, S	2019	Serum Bile Acid Levels Before and After Sleeve Gastrectomy and Their Correlation with Obesity-Related Comorbidities.	Non-extractable or insufficient data
Depommier, C	2019	Supplementation with Akkermansia muciniphila in overweight and obese human volunteers: a proof-of-concept exploratory study	No weight loss interventions
Dong, T	2023	Microbial changes from bariatric surgery alters glucose-dependent insulinotropic polypeptide and prevents fatty liver disease.	No assessment of outcome of interest
Ferolla, S	2016	Beneficial Effect of Synbiotic Supplementation on Hepatic Steatosis and Anthropometric Parameters, But Not on Gut Permeability in a Population with Nonalcoholic Steatohepatitis.	No weight loss interventions
Firouzjaei, A	2016	Comparative evaluation of the therapeutic effect of metformin monotherapy with metformin and acupuncture combined therapy on weight loss and insulin sensitivity in diabetic patients	No assessment of outcome of interest
Gan, J	2023	Action Mechanisms of Metformin Combined with Exenatide and Metformin Only in the Treatment of PCOS in Obese Patients	No assessment of outcome of interest
Gomes, A	2017	The additional effects of a probiotic mix on abdominal adiposity and antioxidant Status: A double-blind, randomized trial.	No weight loss interventions

Guo, J	2022	Fibroblast Growth Factor 19 and Fibroblast Growth Factor 21 Regulation in Obese Diabetics, and Non-Alcoholic Fatty Liver Disease after Gastric Bypass.	No assessment of outcome of interest
Gutiérrez-Repiso, C	2019	Effect of Synbiotic Supplementation in a Very-Low-Calorie Ketogenic Diet on Weight Loss Achievement and Gut Microbiota: A Randomized Controlled Pilot Study.	No assessment of outcome of interest
Haluzíková, D	2013	Laparoscopic sleeve gastrectomy differentially affects serum concentrations of FGF-19 and FGF-21 in morbidly obese subjects.	No assessment of outcome of interest
Haufe, S	2017	Branched-chain amino acid catabolism rather than amino acids plasma concentrations is associated with diet-induced changes in insulin resistance in overweight to obese individuals.	No assessment of outcome of interest
Heianza, Y	2022b	Changes in bile acid subtypes and long-term successful weight-loss in response to weight-loss diets: The POUNDS lost trial.	Non-extractable or insufficient data
Heianza, Y	2018	Changes in Gut Microbiota-Related Metabolites and Long-term Successful Weight Loss in Response to Weight-Loss Diets: The POUNDS Lost Trial.	Non-extractable or insufficient data
Heianza, Y	2022a	Changes in circulating bile acid subtypes in response to weight-loss diets are associated with improvements in glycemic status and insulin resistance: The POUNDS Lost trial.	Same population as other study
Heianza, Y	2024	Changes in bile acid subtypes and improvements in lipid metabolism and atherosclerotic cardiovascular disease risk: the Preventing Overweight Using Novel Dietary Strategies (POUNDS Lost) trial.	Same population as other study
Hron, B	2017	Hepatic, adipocyte, enteric and pancreatic hormones: Response to dietary macronutrient composition and relationship with metabolism	No assessment of outcome of interest
Huang, H	2021	Metabolomics Analysis on Obesity-Related Obstructive Sleep Apnea After Weight Loss Management: A Preliminary Study.	Non-extractable or insufficient data
Huang, H	2019	Bile acid and fibroblast growth factor 19 regulation in obese diabetics, and non-alcoholic fatty liver disease after sleeve gastrectomy	No assessment of outcome of interest
Hughes, T	2013	Ascending dose-controlled trial of beloranib, a novel obesity treatment for safety, tolerability, and weight loss in obese women.	No assessment of outcome of interest
Jansen, P	2011	Alterations of hormonally active fibroblast growth factors after Roux-en-Y gastric bypass surgery.	No assessment of outcome of interest
Johnston, C	2006	Ketogenic low-carbohydrate diets have no metabolic advantage over nonketogenic low-carbohydrate diets	No assessment of outcome of interest
Juárez-Fernández, M	2021	Long-Term Effects of Bariatric Surgery on Gut Microbiota Composition and Faecal Metabolome Related to Obesity Remission.	No assessment of outcome of interest
Kamphuis, M	2003	The effect of conjugated linoleic acid supplementation after weight loss on body weight regain, body composition, and resting metabolic rate in overweight subjects.	No assessment of outcome of interest
Kaválková, P	2016	Endocrine effects of duodenal-jejunal exclusion in obese patients with type 2 diabetes mellitus.	No assessment of outcome of

			interest
Kavanagh, K	2019	Biomarkers of leaky gut are related to inflammation and reduced physical function in older adults with cardiometabolic disease and mobility limitations.	No assessment of outcome of interest
Kellerer, T	2019	Impact of Laparoscopic Sleeve Gastrectomy on Gut Permeability in Morbidly Obese Subjects.	No assessment of outcome of interest
Khakimov, B	2016	New Nordic Diet versus Average Danish Diet: a Randomized Controlled Trial Revealed Healthy Long-Term Effects of the New Nordic Diet by GC-MS Blood Plasma Metabolomics	Non-extractable or insufficient data
Kjeldsen, S	2023	Markers of Glucagon Resistance Improve With Reductions in Hepatic Steatosis and Body Weight in Type 2 Diabetes	No assessment of outcome of interest
Krotkiewski, M	2001	Value of VLCD supplementation with medium chain triglycerides.	No assessment of outcome of interest
Kunesová, M	2006	The influence of n-3 polyunsaturated fatty acids and very low calorie diet during a short-term weight reducing regimen on weight loss and serum fatty acid composition in severely obese women.	No assessment of outcome of interest
Lee, G	2021	Short-term changes in the serum metabolome after laparoscopic sleeve gastrectomy and Roux-en-Y gastric bypass.	Non-extractable or insufficient data
Li, X	2024	Ketogenic diet-induced bile acids protect against obesity through reduced calorie absorption.	Non-extractable or insufficient data
Lips, M	2014	Calorie restriction and Roux-en-Y gastric bypass have opposing effects on circulating FGF21 in morbidly obese subjects.	No assessment of outcome of interest
Liu, C	2024	New mechanistic insights of anti-obesity by sleeve gastrectomy-altered gut microbiota and lipid metabolism	No assessment of outcome of interest
Liu, X	2012	Metabolic and neuroendocrine responses to Roux-en-Y gastric bypass. I: energy balance, metabolic changes, and fat loss.	No assessment of outcome of interest
Luo, P	2016	Metabolomics Study of Roux-en-Y Gastric Bypass Surgery (RYGB) to Treat Type 2 Diabetes Patients Based on Ultraperformance Liquid Chromatography-Mass Spectrometry.	Non-extractable or insufficient data
Luong, T	2024	A ketogenic diet lowers myocardial fatty acid oxidation but does not affect oxygen consumption: a study in overweight humans.	No assessment of outcome of interest
Luong, T	2023	A ketogenic diet reduces myocardial fatty acid oxidation but does not affect myocardial oxygen consumption	No assessment of outcome of interest
Maghsoodi, N	2017	Elevated fasting and postprandial C-terminal telopeptide after Roux-en-Y gastric bypass.	No assessment of outcome of interest
Mak, T	2020	Bile acid, glucose, lipid profile, and liver enzyme changes in prediabetic patients 1 year after sleeve gastrectomy.	No assessment of outcome of interest

Martins, C	2023	Association between Fat-Free Mass Loss, Changes in Appetite, and Weight Regain in Individuals with Obesity.	No assessment of outcome of interest
Martins, C	2021	Association between ketosis and metabolic adaptation at the level of resting metabolic rate.	No assessment of outcome of interest
Matoulek, M	2014	Post-exercise changes of beta hydroxybutyrate as a predictor of weight changes.	No assessment of outcome of interest
Meckling, K	2004	Comparison of a low-fat diet to a low-carbohydrate diet on weight loss, body composition, and risk factors for diabetes and cardiovascular disease in free-living, overweight men and women.	No assessment of outcome of interest
Mohr, A	2018	Exploratory analysis of one versus two-day intermittent fasting protocols on the gut microbiome and plasma metabolome in adults with overweight/obesity.	Non-extractable or insufficient data
Mueller, N	2021	Metformin Affects Gut Microbiome Composition and Function and Circulating Short-Chain Fatty Acids: A Randomized Trial.	Same population as other study
Narath, S	2016	An Untargeted Metabolomics Approach to Characterize Short-Term and Long-Term Metabolic Changes after Bariatric Surgery.	Non-extractable or insufficient data
Nicolucci, A	2017	Prebiotics Reduce Body Fat and Alter Intestinal Microbiota in Children Who Are Overweight or With Obesity	No weight loss interventions
Noel, O	2021	Roux-en-Y Gastric Bypass Surgery Has Early Differential Effects on Bile Acids and the Levels of Complement Component 3 and Acylation-Stimulating Protein.	Non-extractable or insufficient data
Ocaña-Wilhelmi, L	2021	Gut Microbiota Metabolism of Bile Acids Could Contribute to the Bariatric Surgery Improvements in Extreme Obesity.	No assessment of outcome of interest
Ott, B	2017	Effect of caloric restriction on gut permeability, inflammation markers, and fecal microbiota in obese women.	No assessment of outcome of interest
Palau-Rodriguez, M	2020	Effects of a long-term lifestyle intervention on metabolically healthy women with obesity: Metabolite profiles according to weight loss response.	No assessment of outcome of interest
Palau-Rodriguez, M	2018	Characterization of Metabolomic Profile Associated with Metabolic Improvement after Bariatric Surgery in Subjects with Morbid Obesity.	Non-extractable or insufficient data
Pereira, M	2018	A Randomized Controlled Trial of Dapagliflozin Plus Once-Weekly Exenatide Versus Placebo in Individuals with Obesity and Without Diabetes: Metabolic Effects and Markers Associated with Bodyweight Loss.	No assessment of outcome of interest
Pérez-Rubio, A	2023	Gut Microbiota and Plasma Bile Acids Associated with Non-Alcoholic Fatty Liver Disease Resolution in Bariatric Surgery Patients.	Non-extractable or insufficient data
Perissiou, M	2020	The Effect of an 8 Week Prescribed Exercise and Low-Carbohydrate Diet on Cardiorespiratory Fitness, Body Composition and Cardiometabolic Risk Factors in Obese Individuals: A Randomised Controlled Trial.	No assessment of outcome of interest
Pontiroli, A	1996	Benfluorex in obese noninsulin dependent diabetes mellitus patients poorly controlled by insulin: a double blind study versus placebo.	No assessment of outcome of interest

Porter Star, K	2019	Impact on cardiometabolic risk of a weight loss intervention with higher protein from lean red meat: Combined results of 2 randomized controlled trials in obese middle-aged and older adults.	Non-extractable or insufficient data
Prudêncio, A	2023	Red Meat Intake, Indole-3-Acetate, and Dorea longicatena Together Affect Insulin Resistance after Gastric Bypass.	No assessment of outcome of interest
Ramos-Molina, B	2018	Differential effects of restrictive and malabsorptive bariatric surgery procedures on the serum lipidome in obese subjects.	No assessment of outcome of interest
Ritze, Y	2016	Gastric ghrelin, GOAT, leptin, and leptinR expression as well as peripheral serotonin are dysregulated in humans with obesity.	No assessment of outcome of interest
Roager, H	2019	Whole grain-rich diet reduces body weight and systemic low-grade inflammation without inducing major changes of the gut microbiome: a randomised cross-over trial.	No weight loss interventions
Rodgers, M	2021	Weight Loss Outcomes Among Early High Responders to Exenatide Treatment: A Randomized, Placebo Controlled Study in Overweight and Obese Women	No assessment of outcome of interest
Rohde, U	2016	EndoBarrier gastrointestinal liner. Delineation of underlying mechanisms and clinical effects.	No assessment of outcome of interest
Roza, J	2024	Effect of SophorOx(®) on Oxidative Stress and Body Composition in Individuals with High BMI: A Randomized Controlled Trial.	No weight loss interventions
Salman, M	2019	Study of changes of obesity-related inflammatory cytokines after laparoscopic sleeve gastrectomy.	No assessment of outcome of interest
Samczuk, P	2018	In-and-Out Molecular Changes Linked to the Type 2 Diabetes Remission after Bariatric Surgery: An Influence of Gut Microbes on Mitochondria Metabolism.	No assessment of outcome of interest
Sanchez-Carrillo, S	2021	A body weight loss- and health-promoting gut microbiota is established after bariatric surgery in individuals with severe obesity.	No assessment of outcome of interest
Schmidt, J	2016	Effects of RYGB on energy expenditure, appetite and glycaemic control: a randomized controlled clinical trial.	No assessment of outcome of interest
Scholtz, S	2014	Obese patients after gastric bypass surgery have lower brain-hedonic responses to food than after gastric banding	No weight loss interventions
Schwab, U	2008	Triacylglycerol fatty acid composition in diet-induced weight loss in subjects with abnormal glucose metabolism--the GENOBIN study	No assessment of outcome of interest
Sharifnia, T	2015	Hepatic TLR4 signaling in obese NAFLD.	No weight loss interventions
Shimizu, H	2017	Early Effects of Sleeve Gastrectomy on Obesity-Related Cytokines and Bile Acid Metabolism in Morbidly Obese Japanese Patients.	No assessment of outcome of interest
Solito, A	2021	Supplementation with Bifidobacterium breve BR03 and B632 strains improved insulin sensitivity in children and adolescents with obesity in a cross-over, randomized double-blind placebo-controlled tria	No weight loss interventions

Sowah, S	2020	Changes in Plasma Short-Chain Fatty Acid Levels after Dietary Weight Loss Among Overweight and Obese Adults over 50 Weeks.	Non-extractable or insufficient data
Steinert, R	2013	Bile acids and gut peptide secretion after bariatric surgery: a 1-year prospective randomized pilot trial	No assessment of outcome of interest
Stroeve, J	2016	Weight loss predictability by plasma metabolic signatures in adults with obesity and morbid obesity of the DiOGenes study	No assessment of outcome of interest
Sumithran, P	2013	Ketosis and appetite-mediating nutrients and hormones after weight loss	No assessment of outcome of interest
Trøseid, M	2015	Soluble CD14 is associated with markers of vascular dysfunction in bariatric surgery patients	Same population as other study
Uehira, Y	2023	Impact of the lipase inhibitor orlistat on the human gut microbiota.	Not overweight/obese
Umemura, A	2024	Relationships Between Changes in Serum Ketone Body Levels and Metabolic Effects in Patients with Severe Obesity Who Underwent Laparoscopic Sleeve Gastrectomy	No assessment of outcome of interest
Valenzuela-Vallejo, L	2022	Circulating total and intact GDF-15 levels are not altered in response to weight loss induced by liraglutide or lorcaserin treatment in humans with obesity.	No assessment of outcome of interest
van den Broek, M	2021	Altered bile acid kinetics contribute to postprandial hypoglycaemia after Roux-en-Y gastric bypass surgery.	No weight loss interventions
Vitale, M	2021	Acute and chronic improvement in postprandial glucose metabolism by a diet resembling the traditional Mediterranean dietary pattern: Can SCFAs play a role?	No weight loss interventions
von Schwartzenberg, R	2021	Caloric restriction disrupts the microbiota and colonization resistance	No assessment of outcome of interest
Xue, Q	2024	Circulating MicroRNA-21 and Cardiovascular Risk Reduction in Response to Weight-loss Diets: the Preventing Overweight Using Novel Dietary Strategies (POUNDS Lost) Trial	No assessment of outcome of interest
Yu, H	2015	Chenodeoxycholic Acid as a Potential Prognostic Marker for Roux-en-Y Gastric Bypass in Chinese Obese Patients	Non-extractable or insufficient data
Zhong, Y	2023	The effects of a low carbohydrate diet combined with partial meal replacement on obese individuals	Non-extractable or insufficient data
Zhou, T	2019	Circulating Gut Microbiota Metabolite Trimethylamine N-Oxide (TMAO) and Changes in Bone Density in Response to Weight Loss Diets: The POUNDS Lost Trial.	Same population as other study
Zhou, T	2021	Genetically determined SCFA concentration modifies the association of dietary fiber intake with changes in bone mineral density during weight loss: The Preventing Overweight Using Novel Dietary Strategies (POUNDS LOST) trial.	No assessment of outcome of interest

Figure S1. Summary Plot of Risk of Bias for Randomized Control Trials (Rob2 Tool)

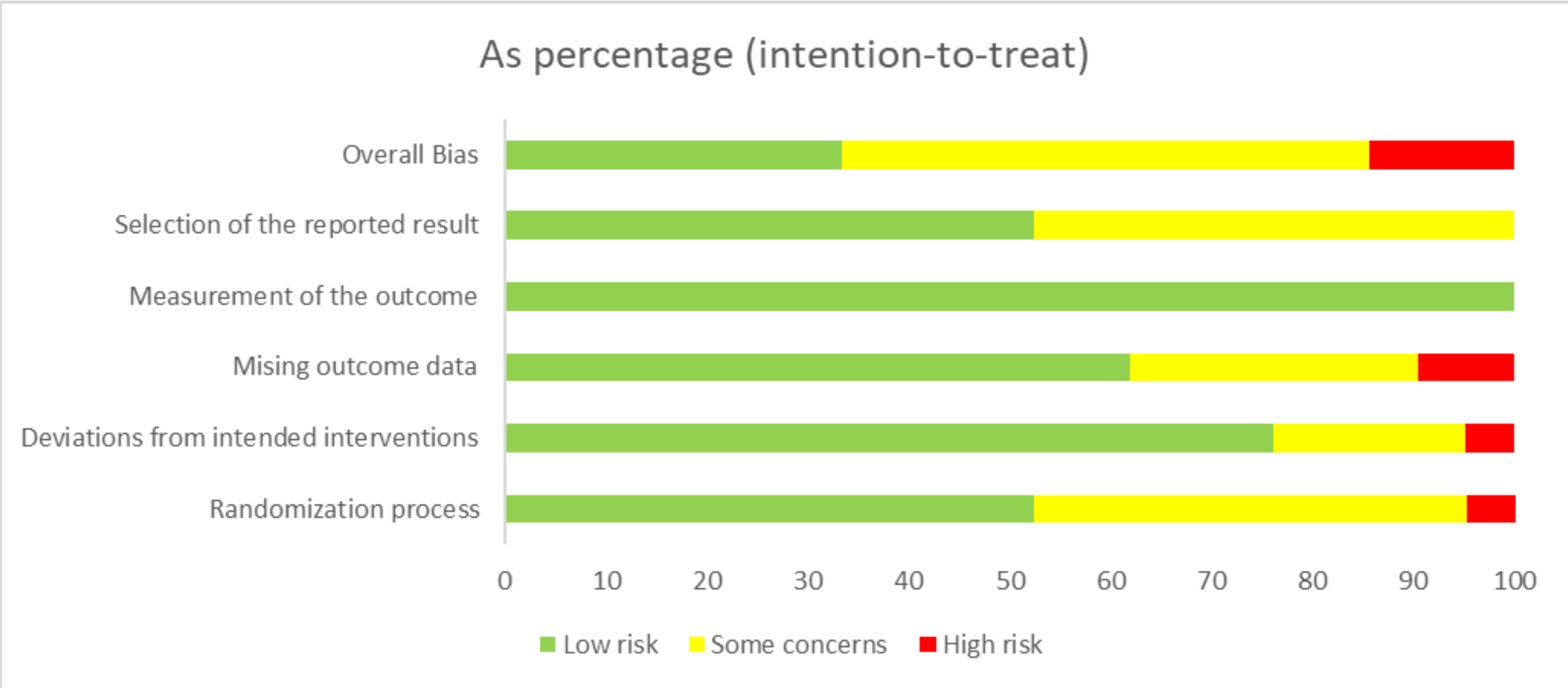

Figure S2. Traffic Light Plot of Risk of Bias for Randomized Control Trials (Rob2 Tool)

Unique ID	Study ID	D1	D2	D3	D4	D5	Overall
1	Alkhatib, 2020	!	+	+	+	!	!
2	Angelidi, 2023	+	+	+	+	+	+
3	Battillo, 2023	!	+	+	+	!	!
4	Diao, 2024 (LPS)	+	+	+	+	+	+
5	Diao, 2024 (TMAO)	+	+	+	+	+	+
6	Erickson, 2019	!	+	!	+	+	!
7	Guevara Cruz, 2024	+	+	+	+	+	+
8	Guo, 2021 (LPS)	+	+	+	+	+	+
9	Guo, 2021 (TMAO)	+	+	+	+	+	+
10	Iversen, 2022	!	+	+	+	+	!
11	Jahansouz, 2016	!	+	-	+	!	-
12	Jianguo, 2021	-	-	+	+	!	-
13	Malin, 2024	!	!	+	+	!	!
14	Nemati, 2018	+	+	-	+	!	-
15	Netto Cândido, 2021	+	+	!	+	+	!
16	Nikard, 2023	+	+	+	+	+	+
17	Risstad, 2017	!	!	!	+	!	!
18	Rooholahzadegan, 2023	+	+	!	+	+	!
19	Sachdev, 2016	+	+	!	+	!	!
20	Tilves, 2022	!	!	!	+	!	!
21	Yang, 2023	!	!	+	+	!	!

+ Low risk
! Guo, 2021 (TMAO)
- High risk

D1 Randomisation process
D2 Deviations from the intended interventions
D3 Missing outcome data
D4 Measurement of the outcome
D5 Selection of the reported result

Figure S3. Summary Plot of Risk of Bias for Non-Randomized Studies of Intervention (Robins-I Tool)

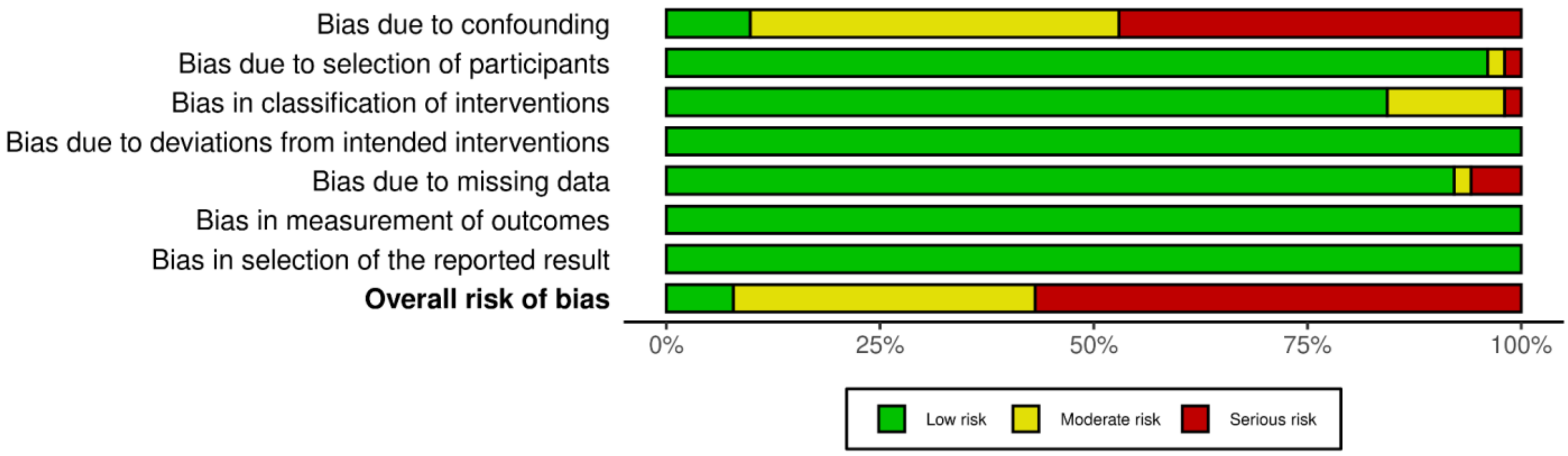

Figure S4. Traffic Light Plot of Risk of Bias for Non-Randomized Studies of Intervention (Robins-I Tool)

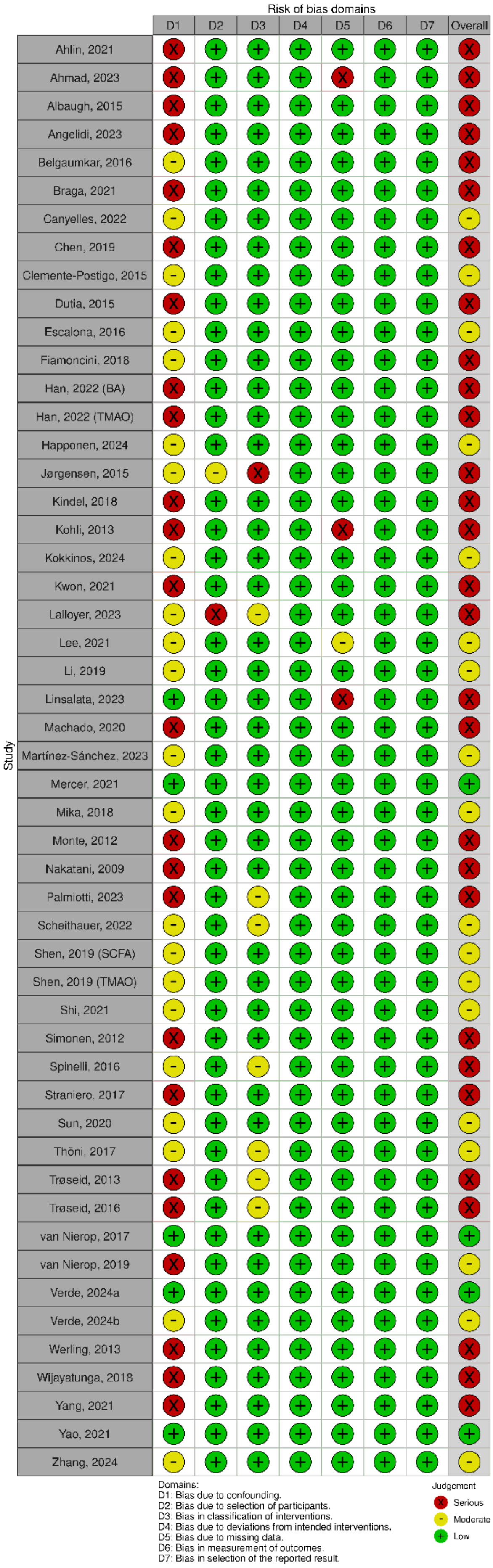

Table S3. Sensitivity and Subgroup Analyses of the Effect of Non-Surgical Weight Loss Interventions on LPS Levels

Sensitivity Analyses	n of studies	SMD (CI 95%)	I^2 (%)	p-value (heterogeneity)
**Δ BMI ≥5%**	4	-0.02 (-1.24, 1.21)	96.1	< 0.0001
**Exclusion of studies with high risk of bias**	7	-0.20 (-0.48, 0.08)	81.6	< 0.0001
**Exclusion of studies with non-dietary interventions or with co-interventions**	5	0.03 (-0.78, 0.83)	92.6	< 0.0001

Legend | LPS = lipopolysaccharide; SMD = Standardized Mean Difference; CI = confidence interval; BMI = Body Mass Index.

Table S4. Sensitivity and Subgroup Analyses of the Effect of Surgical Weight Loss Interventions on LPS Levels

Sensitivity Analyses	n of studies	SMD (CI 95%)	I^2 (%)	p-value (heterogeneity)
**Δ BMI ≥5%**	6	-0.47 (-0.88, -0.07)	82.0	< 0.0001
**Exclusion of studies with high risk of bias**	4	-0.48 (-0.97, 0.02)	86.0	< 0.0001
**RYGB, BPD or DS studies**	6	-0.37 (-0.70, -0.04)	72.9	0.0024

Legend | LPS = lipopolysaccharide; SMD = Standardized Mean Difference; CI = confidence interval; BMI = Body Mass Index; RYGB = Roux-en-Y Gastric Bypass; BPD = biliopancreatic diversion; DS = duodenal switch; SG = sleeve gastrectomy.

Table S5. Sensitivity and Subgroup Analyses of the Effect of Non-Surgical Weight Loss Interventions on TMAO Levels

Sensitivity Analyses	n of studies	SMD (CI 95%)	I^2 (%)	p-value (heterogeneity)
**Δ BMI ≥5%**	4	-0.78 (-1.42, -0.14)	82.6	0.0006
**Exclusion of studies with high risk of bias**	8	-0.56 (-0.90, -0.22)	74.7	0.0002
**Exclusion of studies with non-dietary interventions or with co-interventions**	4	-0.82 (-1.36, -0.28)	80.8	0.0014

Legend | TMAO = trimethylamine N-oxide; SMD = Standardized Mean Difference; CI = confidence interval; BMI = Body Mass Index.

Table S6. Sensitivity and Subgroup Analyses of the Effect of Surgical Weight Loss Interventions on TMAO Levels

Sensitivity Analyses	n of studies	SMD (CI 95%)	I^2 (%)	p-value (heterogeneity)
**Δ BMI ≥5%**	7	0.46 (-0.02, 0.93)	82.1	< 0.0001
**Exclusion of studies with high risk of bias**	5	0.62 (-0.07, 1.30)	86.3	< 0.0001
**RYGB or DS studies**	5	0.81 (0.28, 1.34)	69.4	0.0110
**SG studies**	3	0.14 (-0.15, 0.43)	0.0	0.6228

Legend | TMAO = trimethylamine N-oxide; SMD = Standardized Mean Difference; CI = confidence interval; BMI = Body Mass Index; RYGB = Roux-en-Y Gastric Bypass; DS = duodenal switch; SG = sleeve gastrectomy.

Table S7. Sensitivity and Subgroup Analyses of the Effect of Surgical Weight Loss Interventions on Secondary Bile Acids Levels

Sensitivity Analyses	n of studies	SMD (CI 95%)	I^2 (%)	p-value (heterogeneity)
**Exclusion of studies with high risk of bias**	6	0.42 (0.11, 0.74)	75.0	0.0013
**RYGB, OLGB, BPD/DS or LSG/DJB studies**	11	0.47 (0.29, 0.66)	49.9	0.0297
**RYGB studies only**	8	0.51 (0.34, 0.68)	26.2	0.2193
**SG or LAGB studies**	7	1.47 (-0.85, 3.78)	89.7	< 0.0001
**SG studies only**	4	2.45 (-1.89, 6.79)	94.6	< 0.0001

Legend | SMD = Standardized Mean Difference; CI = confidence interval; BMI = Body Mass Index; RYGB = Roux-en-Y Gastric Bypass; OLGB = omega-loop gastric bypass; LSG/DJB = Laparoscopic sleeve gastrectomy with duodenojejunal bypass; BPD/DS = biliopancreatic diversion with duodenal switch; SG = sleeve gastrectomy; LAGB = laparoscopic adjustable gastric banding.

Figure S3. Effect of Weight Loss Interventions on UDCA Levels

A. Non-Surgical Weight Loss Interventions

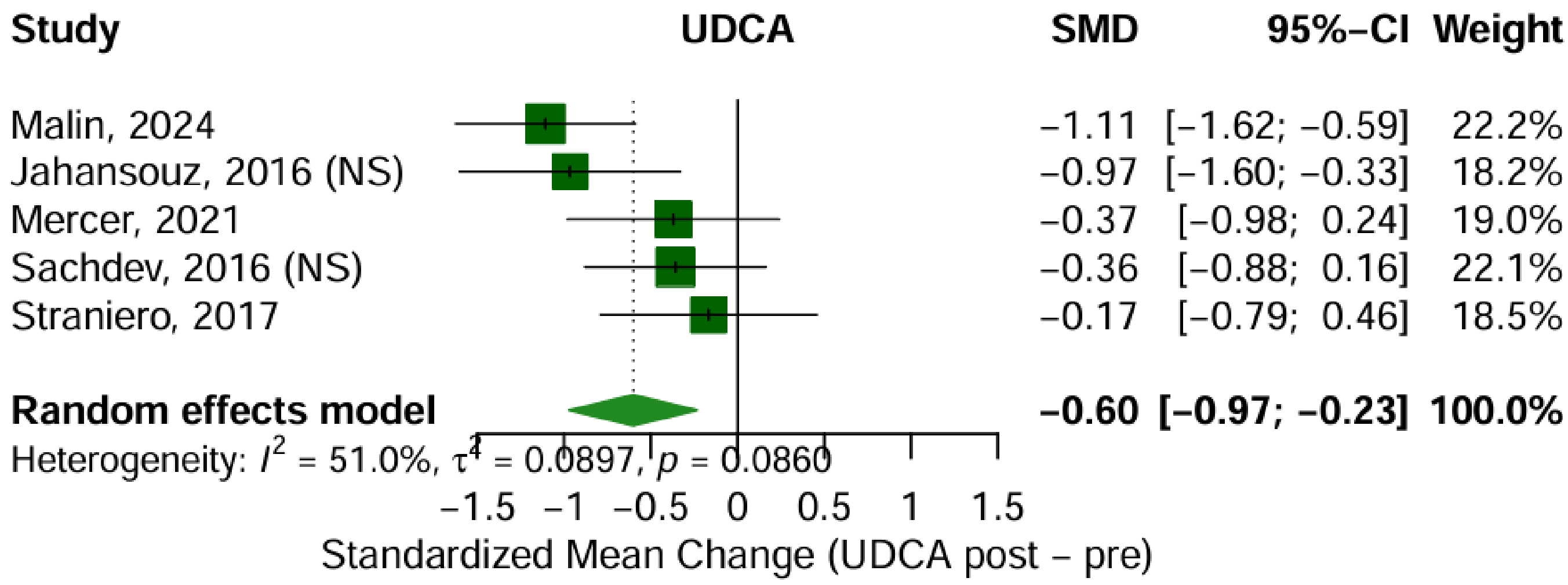

B. Surgical Weight Loss Interventions

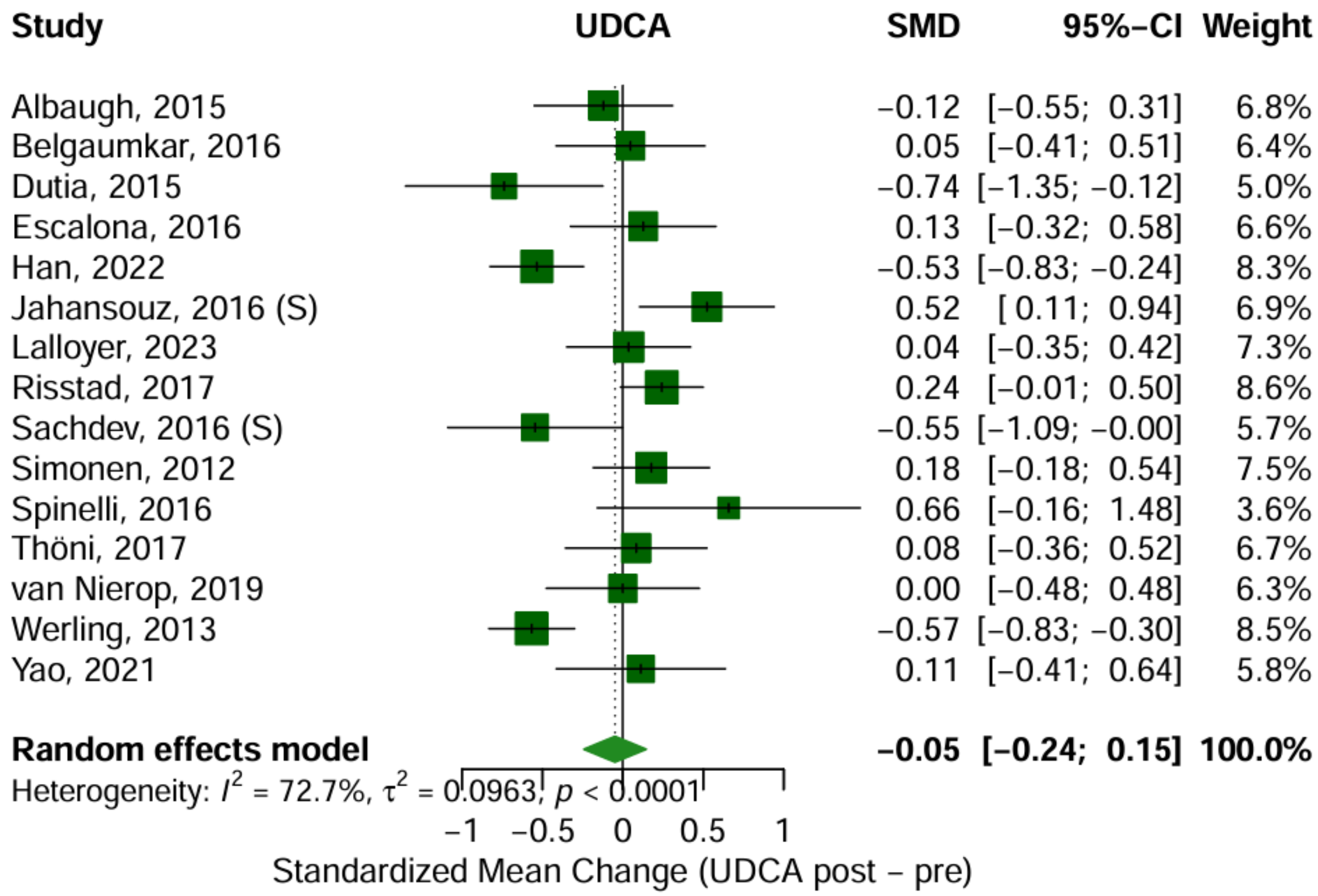

Legend: (NS) = non-surgical; (S) = surgical

Figure S4. Effect of Weight Loss Interventions on LCA Levels

A. **Surgical Weight Loss Interventions**

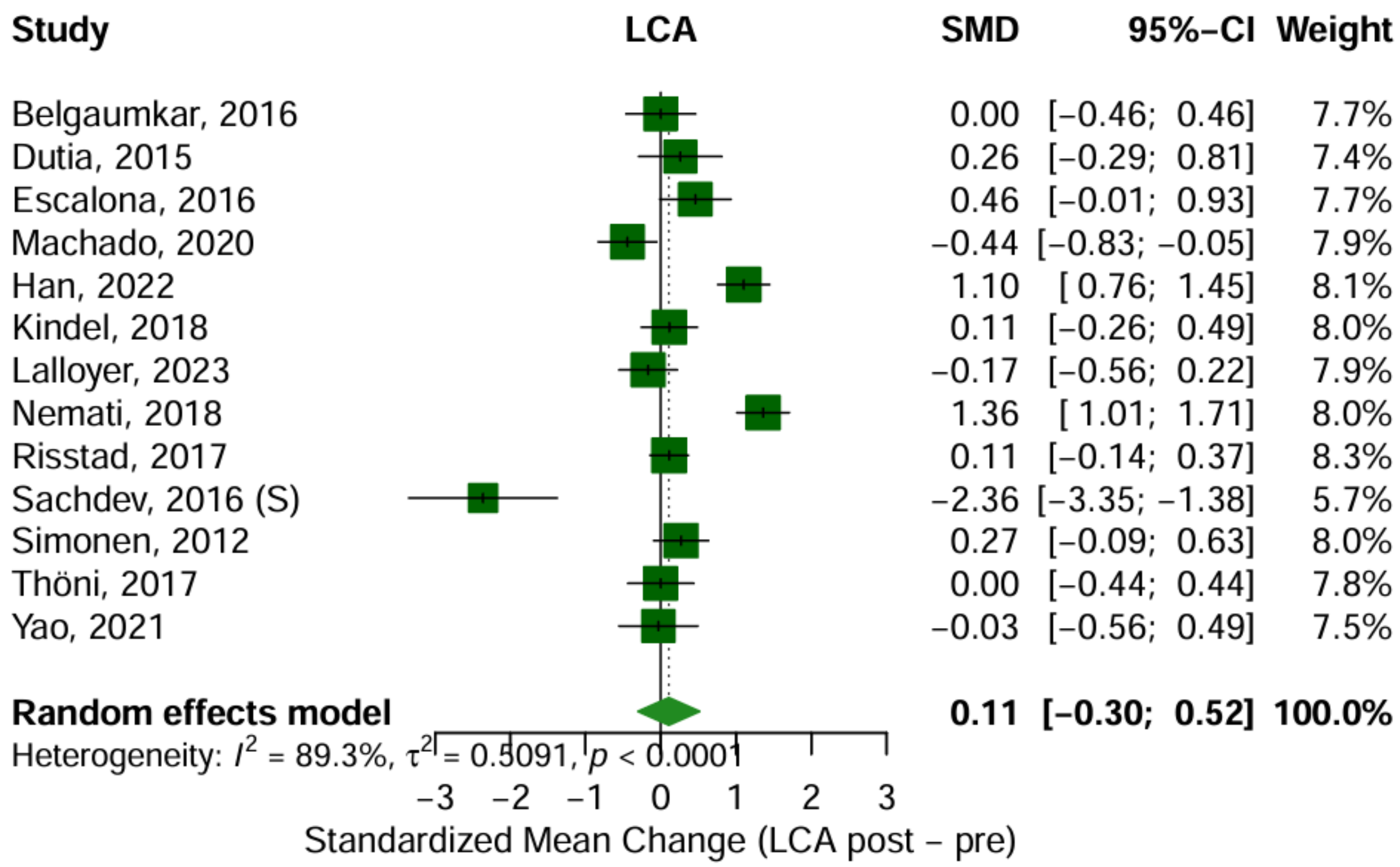

Legend: (S) = surgical;

Figure S5. Effect of Weight Loss Interventions on DCA Levels

A. **Non-Surgical Weight Loss Interventions**

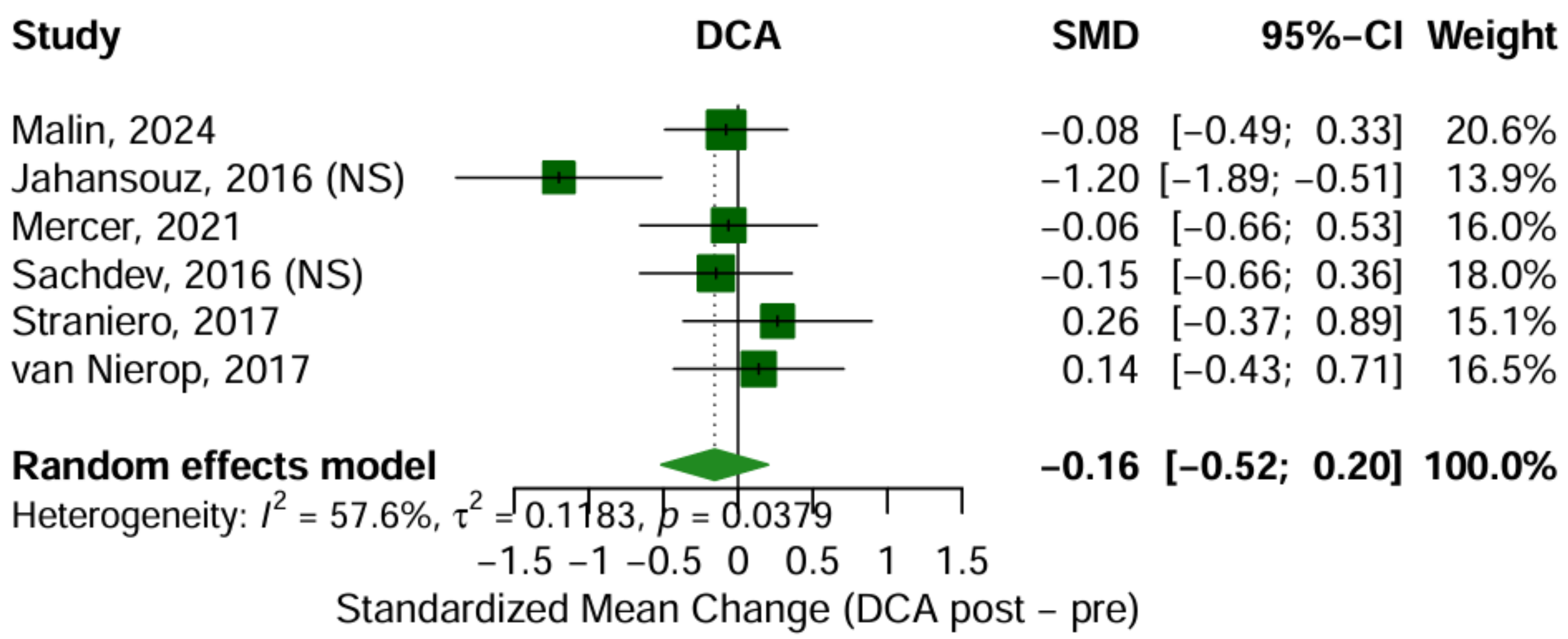

B. **Surgical Weight Loss Interventions**

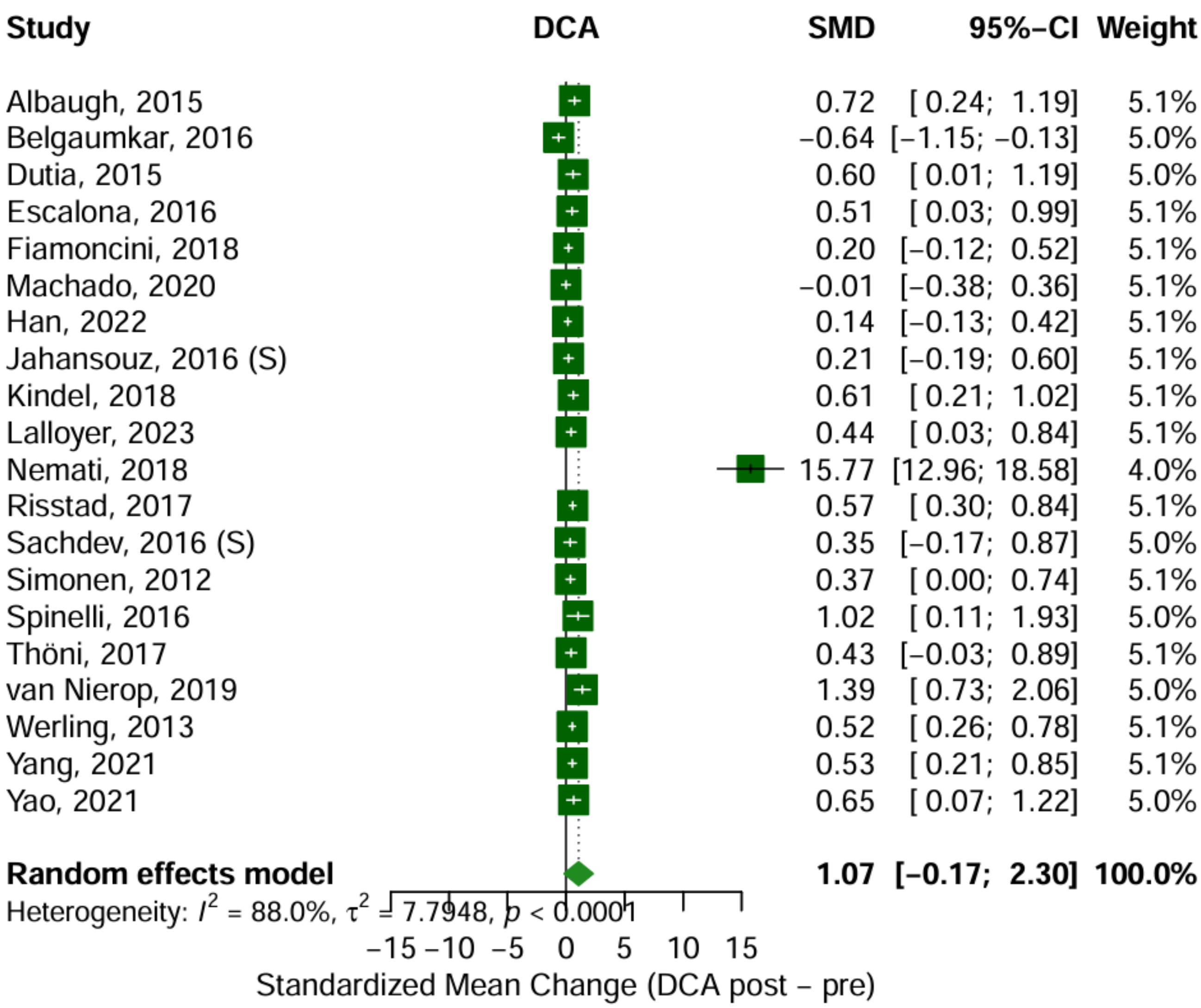

Legend: (NS) = non-surgical; (S) = surgical
